# Combining Metabolomics and Transcriptomics to Reveal the Mechanism of Coloration in Purple and Cream Mutant of Sweet Potato (*Ipomoea batatas* L.)

**DOI:** 10.3389/fpls.2022.877695

**Published:** 2022-05-04

**Authors:** Rong Zhang, Ming Li, Chaochen Tang, Bingzhi Jiang, Zhufang Yao, Xueying Mo, Zhangying Wang

**Affiliations:** ^1^Crops Research Institute, Guangdong Academy of Agricultural Sciences & Key Laboratory of Crop Genetic Improvement of Guangdong Province, Guangzhou, China; ^2^Institute of Biotechnology and Nuclear Technology, Sichuan Academy of Agricultural Sciences, Chengdu, China

**Keywords:** sweet potato, flesh color, roots quality, anthocyanin biosynthesis, phenylalanine metabolism, volatile compounds

## Abstract

Purple sweet potato is considered as a healthy food because of its high anthocyanins. To understand the coloring mechanism and quality change between purple-fleshed sweet potato (cv. Xuzi201) and its cream fleshed mutant (M1001), a combined metabolomic and transcriptomic analysis was performed. The metabolome data showed that 4 anthocyanins, 19 flavones, 6 flavanones, and 4 flavonols dramatically decreased in M1001, while the contents of 3 isoflavones, 3 flavonols, 4 catechins, and 2 proanthocyanins increased. Transcriptomic analyses indicated that the expression of 49 structural genes in the flavonoid pathway and transcription factors (TFs) (e.g., *bHLH2, R2R3-MYB, MYB1*) inducting anthocyanin biosynthesis were downregulated, but the repressor *MYB44* was upregulated. The *IbMYB1-2* gene was detected as a mutation gene in M1001, which is responsible for anthocyanin accumulation in the storage roots. Thus, the deficiency of purple color in the mutant is due to the lack of anthocyanin accumulation which was regulated by *IbMYB1*. Moreover, the accumulation of starch and aromatic volatiles was significantly different between Xuzi201 and M1001. These results not only revealed the mechanism of color mutation but also uncovered certain health-promoting compounds in sweet potato.

## Introduction

Sweet potato (*Ipomoea batatas* L.), a member of Convolvulaceae family, is the fifth most essential food crop on a fresh weight basis (Zhu et al., 2022). The annual global production of sweet potato is ~90 million tons of fresh storage roots (FAOSTAT, 2021). Sweet potato has been used for a wide range of applications, such as table use, processed foods, starch production, and animal feed (Wang et al., 2016; de Albuquerque et al., 2019). The tuberous roots of sweet potato accumulated natural pigments and exhibited different flesh colors, such as white, cream, yellow, orange, and purple. Sweet potato is a rich source of carbohydrates, dietary fiber, β-carotene, minerals, and bioactive compounds; the content and the composition of sweet potato depend on cultivars with different flesh colors (Wang et al., 2018a). Purple-fleshed sweet potato (PFSP) not only contains the nutrients of ordinary sweet potatoes but also possesses an attractive purple color and a high antioxidant activity since it accumulated large amounts of anthocyanins (Bovell Benjamin, 2007; Steed and Truong, 2008). Several studies have reported that anthocyanins isolated from PFSP have hypoglycemic effects (Jang et al., 2019), anti-cancer activity (Asadi et al., 2017), anti-obesity effects (Kim et al., 2020), cardioprotective effect, and anti-atherogenic potential (Alam, 2021). Anthocyanins extracted from PFSP are widely used as dietary supplements and additives in the food industry. However, the consumer surveys showed that PFSPs were characterized by dense and firm texture, significant fibrous visual and lack of moistness, sweet taste, brown sugar or floral flavor, and aroma compared with orange and yellow sweet potatoes (Leksrisompong et al., 2012). The taste texture and flavor were directly related to the tuberous roots' quality characters such as dry matter content, starch content and structure, soluble sugar content, and aroma volatile compounds. Therefore, identifying the chemical and genetic composition of sweet potato is crucial for understanding the color formation and the tuberous root quality, and for uncovering the nutritional value and increasing the commercial benefit of sweet potato.

For a long time, a wide variety of studies have been focused on anthocyanin chemical structure and biosynthesis. The main anthocyanins in sweet potato were mono- and diacylated derivatives of cyanidins and peonidins (Montilla et al., 2010) that were synthesized *via* the flavonoid pathway, which has been widely studied in a variety of plants like Arabidopsis (Lepiniec et al., 2006), strawberry (Aharoni et al., 2001), *Passiflora edulis Sims* (Qiu et al., 2020), and purple wheat (Wang et al., 2021). In sweet potato, the key structural genes related to the flavonoid biosynthetic pathway, such as phenylalanine ammonia lyase (*IbPAL*) (Yu et al., 2021), chalcone isomerase (*IbCHI*) (Guo et al., 2015), dihydrokaempferol reductase (*IbDHKR*) (Liu et al., 2017), dihydroflavonol 4 reductase (*IbDFR*) (Wang et al., 2013), anthocyanidin synthase (*IbANS*) (Zhou et al., 2009), flavonoid 3′-hydroxylase (*IbF3'H*) (Zhou et al., 2012), anthocyanidin 3-O-glucoside-2″-O-glucosyltransferase (*Ib3GGT*) (Wang et al., 2018b), and UDP-glucose: flavonoid 3-O-glucosyltransferase (*IbUF3GT*) (Hu et al., 2016), have been isolated and verified with gene function illustration. In addition to structural genes, many transcription factors (TFs) have also been reported to regulating the expression of flavonoid biosynthetic genes. *IbMYB1* belongs to the R2R3-MYB that are involved in anthocyanin biosynthesis and was isolated and specifically expressed in the PFSP roots. Expression of *IbMYB1* alone was sufficient for the induction of all structural anthocyanin genes and anthocyanin accumulation in the flesh of tuberous roots (Mano et al., 2007; Park et al., 2015; Zhang D. et al., 2021). *IbMYB1*-2a/b was identified as a mutation site in the white flesh mutation of purple sweet potato, which is responsible for anthocyanin accumulation in the storage roots (Tanaka et al., 2012), but the expression of *IbMYB1* in all varieties is not necessary (Kim et al., 2010; Li et al., 2019; Zhang et al., 2020). Recently, an anthocyanin biosynthesis repressor *IbMYB44* was cloned in sweet potato, and it competitively inhibited the formation of *IbMYB340-IbbHLH2-IbNAC56a/b* regulatory complex (Wei et al., 2020).

Although some structural and regulatory genes have been identified affecting the coloration of PFSP, the chemical compositions, especially the non-anthocyanin flavonoids components and volatile compounds related to the nutrition and flavor quality and the underlying potential regulatory mechanism remain unclear. Liquid chromatography-tandem mass spectrometry (LC–MS/MS)-based widely targeted metabolomics analysis is a rapid and highly sensitive method for detecting as many metabolites as possible and is widely used in different areas (Chen et al., 2013). Headspace solid-phase microextraction (HP-SPME) coupled with gas chromatography-mass spectrometry (GC-MS) has been widely used to investigate the volatile compounds in plants including sweet potato (Zhang et al., 2021; Zhang R. et al., 2021). In recent years, combined analysis of transcriptomics (RNA-Seq) and metabolomics has been widely used to identify and screen metabolites and related genes underlying the coloration and quality formation in plants, such as Longan (Yi et al., 2021), *Ziziphus jujuba* Mill. (Zhang Q. et al., 2020), and apricot fruit (Zhou et al., 2021). However, the metabolites and genes related to sweet potato coloration and quality formation were not clear. In this study, one PFSP cultivar Xuzi201 and its cream flesh mutant were thoroughly investigated by using transcriptomics and metabolomics to identify and quantify non-volatile and volatile metabolites and gene expression changes in the tuberous roots. These results greatly extend our understanding of the molecular mechanism of anthocyanin accumulation in sweet potato and provide a reference for evaluating the nutritive value to inform future breeding strategies.

## Materials and Methods

### Plant Materials

**Figure 1:**
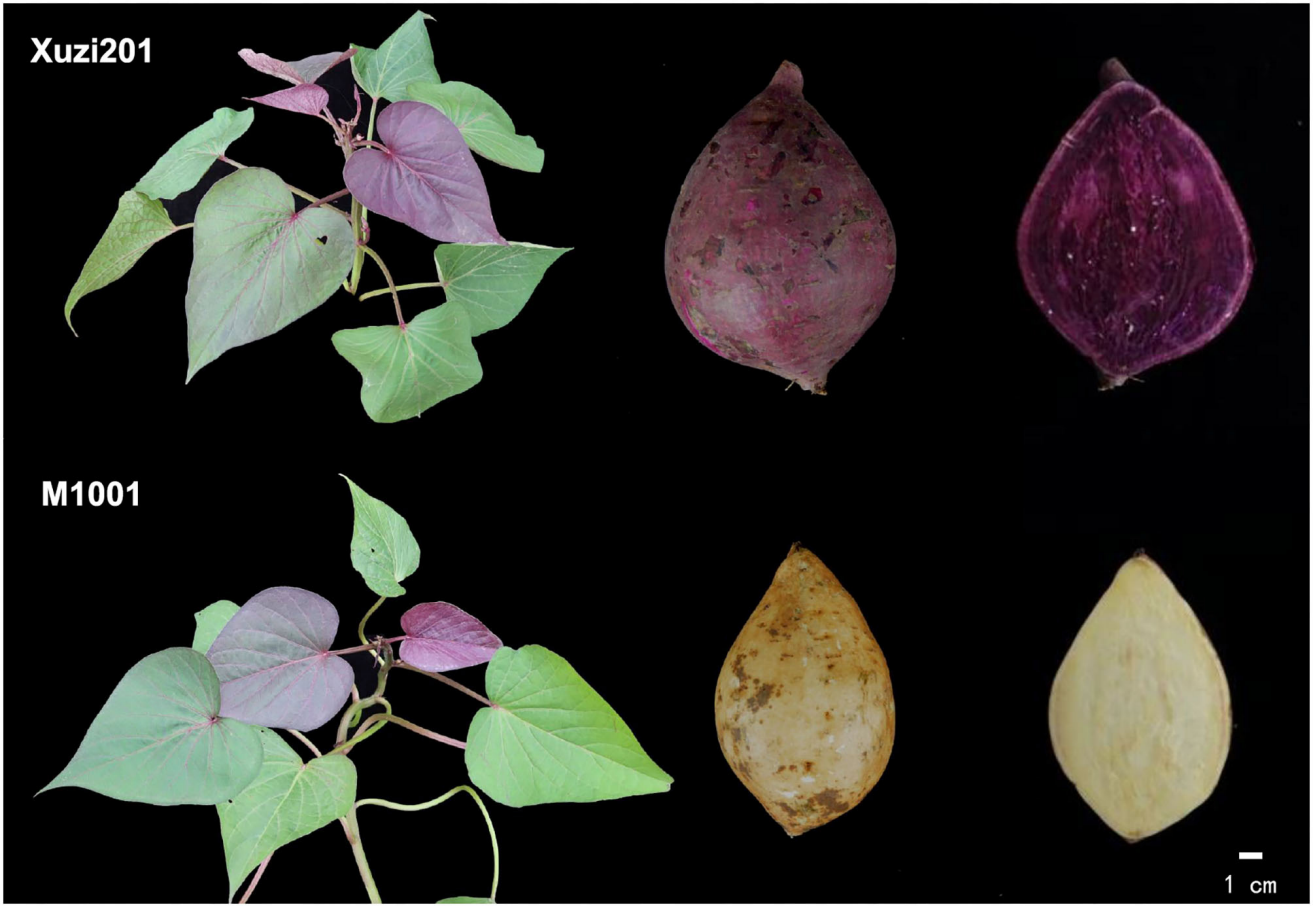
Overview of aboveground parts, tuberous roots, and root fleshes from Xuzi201 and M1001.

Two sweet potato cultivars, namely, Xuzi201 and M1001, were selected for the study (Figure 1). Xuzi201 was a PFSP cultivar, which was bred by Xuzhou Sweet Potato Research Center, China. M1001 was its mutant with cream root skin and cream flesh color, which was selected by the Institute of Biotechnology and Nuclear Technology, Sichuan Academy of Agricultural Sciences, Chengdu, Sichuan, China, and obtained by a total dose of 5 Gy 60 Co γ-ray radiation treatment with 1 Gy/min. The agronomic characteristics of M1001 in the field were uniform, and the genetic characteristics were stable and consistent for more than 10 years.

Xuzi201 and M1001 were cultivated in a randomized field plot according to the standard agricultural practices at the Baiyun Experimental Station (23°23'N, 113°26'E; 20 m above sea level) of Guangdong Academy of Agricultural Sciences, Guangzhou, China. They were planted in May, transplanted in July, and harvested after 120 days. Ten medium-sized tuberous roots were collected, washed with distilled water, air-dried, cut into shreds, combined and blended thoroughly, then frozen in liquid nitrogen, and stored at −80°C for subsequent analysis.

### Analysis of Physiological Parameters

Total starch content and amylose content were measured using a Total Starch Assay Kit (Megazyme, Ireland) and an Amylose/Amylopectin Assay Kit (Megazyme, Ireland) according to the manufacturer's instructions, respectively.

Total flavonoids content was determined using the colorimetric method as described by Shekhar et al. (2015) with modifications. Briefly, 0.1 g of freeze-dried sample was extracted with 10 ml of 70% aqueous ethanol on ultrasonic wave treatment for 30 min. The extract was centrifuged at 5,000 r/min for 10 min at 4°C. The alcoholic tuber extract was diluted to a final volume of 25 ml with 70% aqueous ethanol. Then, 5 ml of the extract solution was mixed with 0.3 ml of 5% sodium nitrite and stood for 5 min at room temperature (26°C). Subsequently, 0.3 ml of 10% aluminum nitrate was added and allowed to stand for 6 min. Furthermore, 4 ml of 1 M sodium hydroxide was added, and the solution was finally added up to 10 ml with 70% aqueous ethanol. The absorbance was determined by spectrophotometer at 510 nm, and the results were expressed as mg/g rutin equivalents on a dry weight basis (mg RE per g DW).

Total anthocyanin content was measured using the pH-differential method described by Steed and Truong (2008) and calculated as cyanidin 3-glucoside using an extinction coefficient (ε) of 26,900 L per cm-mol and molecular weight of 449.2. The total anthocyanins were reported as milligrams anthocyanins per 100 g fresh weight (mg cyanidin-3-glucoside per 100 g FW).

### Metabolome Analysis

A widely targeted metabolomics strategy was used to determine the metabolites in the roots of sweet potatoes. Metabolite extraction, detection, identification, and quantification were carried out according to the LC-MS methods described by Chen et al. (2013). Briefly, the freeze-dried tuber samples were used for LC-MS analysis and crushed using a mixer mill (MM 400, Retsch) with a zirconia bead for 1.5 min at 30 Hz; 100 mg powder was weighed and extracted overnight with 1.0 ml 70% aqueous methanol at 4°C. Following centrifugation at 10,000 g for 10 min, the extracts were absorbed (CNWBOND Carbon-GCB SPE Cartridge, 250 mg, 3 ml; ANPEL, Shanghai, China) and filtrated (SCAA-104, 0.22 μm pore size; ANPEL, Shanghai, China) before LC-MS analysis. The sample extracts were analyzed using an LC-ESI-MS/MS system [HPLC, Shim-pack UFLC SHIMADZU CBM30A system, Kyoto, Japan; MS, Applied Biosystems 4500 Q TRAP, equipped with an Agilent SB-C18 column (1.8 μm, 2.1 × 100 mm, Foster City, CA)]. The mobile phase consisted of pure water containing 0.04% acetic acid (solvent A) and acetonitrile containing 0.04% acetic acid (solvent B). Sample measurements were performed using the following gradient program: 0 min, 5% B; 0–11 min, linear gradient increase to 95% B, 11–12 min, 95% B; 12.0–12.10 min, decrease to 5% B; 12.10–15 min, 5% B. The column oven was set to 40°C, and the injection volume was 5 μl. The effluent was alternatively connected to an ESI-triple quadrupole-linear ion trap (QTRAP). The mixture of all the sample extracts was used as the quality control to monitor the technical reproducibility. Metware database (MWDB) together with the public databases was used to annotate the metabolites. Quantifications of metabolites were conducted using multiple reaction monitoring. The differentially accumulated metabolites (DAMs) between Xuzi201 and M1001 were determined based on the variable importance in projection (VIP) ≥ 1 and Log_2_FC ≥1 or Log_2_FC ≤-1.

### Identification and Quantification of Volatile Compounds

Volatile compounds were determined in a similar way as described in Zhang R. et al. (2021) with modifications. The stored samples of two cultivars were separately grounded in a liquid nitrogen grinder (A10 basic, IKA, Staufen, Germany); 1 g of powder was stored in a 20 ml headspace vial with 1 ml saturated NaCl solution added. Then, 1 μl of decanoic acid, ethyl ester solution (100 μg/ml, CAS: 110-38-3) was also added, and the mixture was gently homogenized.

The headspace vial was incubated for 30 min at 80°C, and then, the volatile compounds were extracted by an SPME device with DVB/CAR/PDMS fiber (Supelco, Bellefonte, PA) for 30 min at the same temperature. The fiber head was inserted into the GC-MS inlet and desorbed at 250°C for 5 min. Each sample was analyzed three times using headspace solid-phase microextraction.

For GC–MS/MS analysis, an 8890-5977B system (Agilent Technologies, Santa Clara, CA) equipped with a DB-5 ms ultra-inert capillary column (60 m × 0.32 mm × 0.25 μm) was used in the splitless injection mode with high-purity helium (99.999%) flow at 1.0 ml/min. The temperature of the GC injector was maintained at 250°C, and the following column temperature program was employed: initial temperature, 35°C (hold for 2 min); increased to 190°C at a rate of 5°C/min (held for 1 min); and increased to 250°C at a rate of 20°C/min (held for 2 min), solvents delay 7 min.

Chromatograms and mass spectra were analyzed using the Enhanced ChemStation software (Agilent Technologies, CA, USA). Identification of volatile compound tentative was achieved by matching the mass spectra with the data system library (NIST 2017) and linear retention index (RI) sourced from NIST Standard Reference Database. Quantitative data for each identified compound were obtained by comparing to the internal standard. All the analyses were repeated three times.

### RNA Extraction, Sequencing, and RNA-Seq Analysis

For transcriptome sequencing, six libraries representing the collected tuberous root samples of Xuzi201 and M1001 (three replicates of each) were constructed. Total RNA was extracted using TaKaRa MiniBEST Plant Extraction Kit (TaKaRa, Beijing, China) according to the manufacturer's instructions. RNA contamination and RNA integrity number (RIN) were monitored on 1% agarose gels and the Agilent 2100 Bioanalyzer system (Agilent Technologies, CA, USA), respectively. A total amount of 3 μg RNA per sample was used as input material for the RNA sample preparations. Sequencing libraries were generated using NEBNext® Ultra™ RNA Library Prep Kit for Illumina® (NEB, USA) following the manufacturer's recommendations, and index codes were added to attribute sequences to each sample. The libraries were then sequenced on an Illumina Novaseq platform, and paired-end reads were generated. Clean reads were extracted with base-pair qualities in the *Q* ≥10 using Perl scripts and sequentially assembled to obtain the Unigene library using Trinity. Then, the clean reads of samples were mapped with the Unigene library. The unigene function was annotated according to the following databases: Nr (NCBI non-redundant protein sequences); Nt (NCBI non-redundant nucleotide sequences); Pfam (Protein family); KOG/COG (Clusters of Orthologous Groups of proteins); Swiss-Prot; KO (Kyoto Encyclopedia of Genes and Genomes (KEGG) Ortholog database); and GO (Gene Ontology). Fragments per kilobase of transcript per million mapped reads (FPKM) were used for transcription or quantification of gene expression levels. An absolute Log_2_ (fold change) ≥1 and a false discovery rate (FDR) < 0.05 were used as thresholds for the identification of differentially expressed genes (DEGs) using DESeq2 R package (v1.34.0) (Love et al., 2014). GO enrichment analysis was performed using the GOSeq R package (v1.46.0, corrected *p* < 0.05). Pathway analysis was performed to elucidate the significant pathways of DEGs using KEGG (www.genome.jp/kegg). The raw transcriptomic data have been uploaded to the National Genomics Data Center [Home - National Genomics Data Center (cncb.ac.cn)], and the BioProject ID is PRJCA008065.

### Amplification of *IbMYB1-2* Maker Fragment

*IbMYB1-2a/2b* fragments were amplified in the 20 μl mixture containing 2 × Taq PCR MasterMix II (TIANGEN BIOTECH, Beijing, China), 5 μM foreword primer, 5 μM reverse primer, and 50 ng cDNA under the following program: 94°C for 3 min; 35 cycles of 94°C for 15 s, 54°C for 30 s, and 72°C for 45 s; and 72°C for 5 min. The primers were used according to the study by Tanaka et al. (2012) and Zhang D. et al. (2021) and are shown in Supplementary Table S1.

### qRT-PCR Analysis

Total RNA was isolated as described for RNA-sequencing analysis. First-strand cDNA was synthesized using FastKing gDNA Dispelling RT SuperMix (TIANGEN, Beijing China). All reactions were performed using the SsoFast Eva Green Supermix kit (Bio-Rad) in a total sample volume of 10 μl (5 μl of SsoFast EvaGreen supermix, 1 μl of Forward primer, 1 μl of Reverse primer, 3 μl of cDNA template). Quantitative real-time PCR (RT-qPCR) analysis was carried out using the Bio-Rad CFX96™ Real-Time System (Hercules, CA, United States). The program of two-step real-time RT-PCR was 95°C for 30 s, followed by 40 cycles of 95°C for 5 s, and 60°C for 10 s (Pfaffl, 2001). Each sample was analyzed in three technical replicates. The relative expression level of mRNAs was normalized to that of internal control *Ibactin* (AY905538) by using the 2^Δ*ΔCt*^ cycle threshold method. The primers are shown in Supplementary Table S1.

### Statistical Analysis

Each cultivar was tested in triplicate to ensure the reliability of the experimental results. All data were analyzed using SPSS statistics 24 and expressed as mean ± SD. One-way ANOVA was implemented and followed by Tukey's significant difference test with significance set at *P* < 0.05. The unsupervised principal component analysis (PCA) score plot, Volcano plots, KEGG enrichment plot, OPLS-DA model plots, and heatmap were carried out using the *prcomp, ggplot2* (v3.3.5), and *pheatmap* (v1.0.12) packages of R.

## Results

### Phenotypic Characterization of Purple and Cream Mutant of Sweet Potato

A total of 24 morphological descriptor characteristics, namely, 2 vine tips, 2 immature leaf, 10 leaves, 8 vine, and 2 roots characteristics, of Xuzi201 and M1001 were characterized according to the study by Zhang and Fang (2006). No significant morphological difference was identified between Xuzi201 and its mutant M1001, except for skin and flesh color (Figure 1, Supplementary Table S2). To further define whether there is a quality difference between them, five major quality-related parameters (i.e., dry rate, starch, amylose, flavonoids, and anthocyanin) were detected in the wild type and the mutant. As shown in Table 1, the dry matter content of Xuzi201 was 32.65% ± 0.40, while that of M1001 significantly decreased, with the dry matter content 27.86% ± 0.60. The starch content also decreased in M1001 than in Xuzi201, with the starch content value of 12.73% ± 0.12 and 19.64% ± 0.9, respectively. Among the total starch, the percentage of amylose content of Xuzi201 was similar to M1001, with the amylose content of 11.78% ± 1.68 and 10.90% ± 0.39, respectively. The contents of flavonoids and anthocyanins were closely related to the color of sweet potato. The flavonoids content of Xuzi201 was 6.7 ± 0.26 mg/g DW, which was significantly higher than that of M1001 0.4 ± 0.22 mg/g DW. The anthocyanins content of Xuzi201 was 26.21 ± 1.41 mg/100 g FW, while no anthocyanins was detected in M1001 using the pH-differential method.

**Table 1:** Contents of quality parameters of Xuzi201 and M1001.

	**Xuzi201**	**M1001**	***P*** **-value**
Dry matter (%)	32.65 ± 0.40	27.86 ± 0.60	<0.001
Starch (%, FW)	19.64 ± 0.79	12.73 ± 0.12	0.002
Amylose (%)	11.78 ± 1.68	10.90 ± 0.39	0.051
Flavonoid (mg/g, DW)	6.7 ± 0.26	0.4 ± 0.22	<0.001
Anthocyanin (mg/100±g, FW)	26.21 ± 1.41	−	

*Data were expressed as average ± standard variation*.

*The ANOVA was performed, and p < 0.05 was considered a significant difference*.

### Non-volatile Metabolome Profiles of Purple and Cream Mutant of Sweet Potato

**Figure 2:**
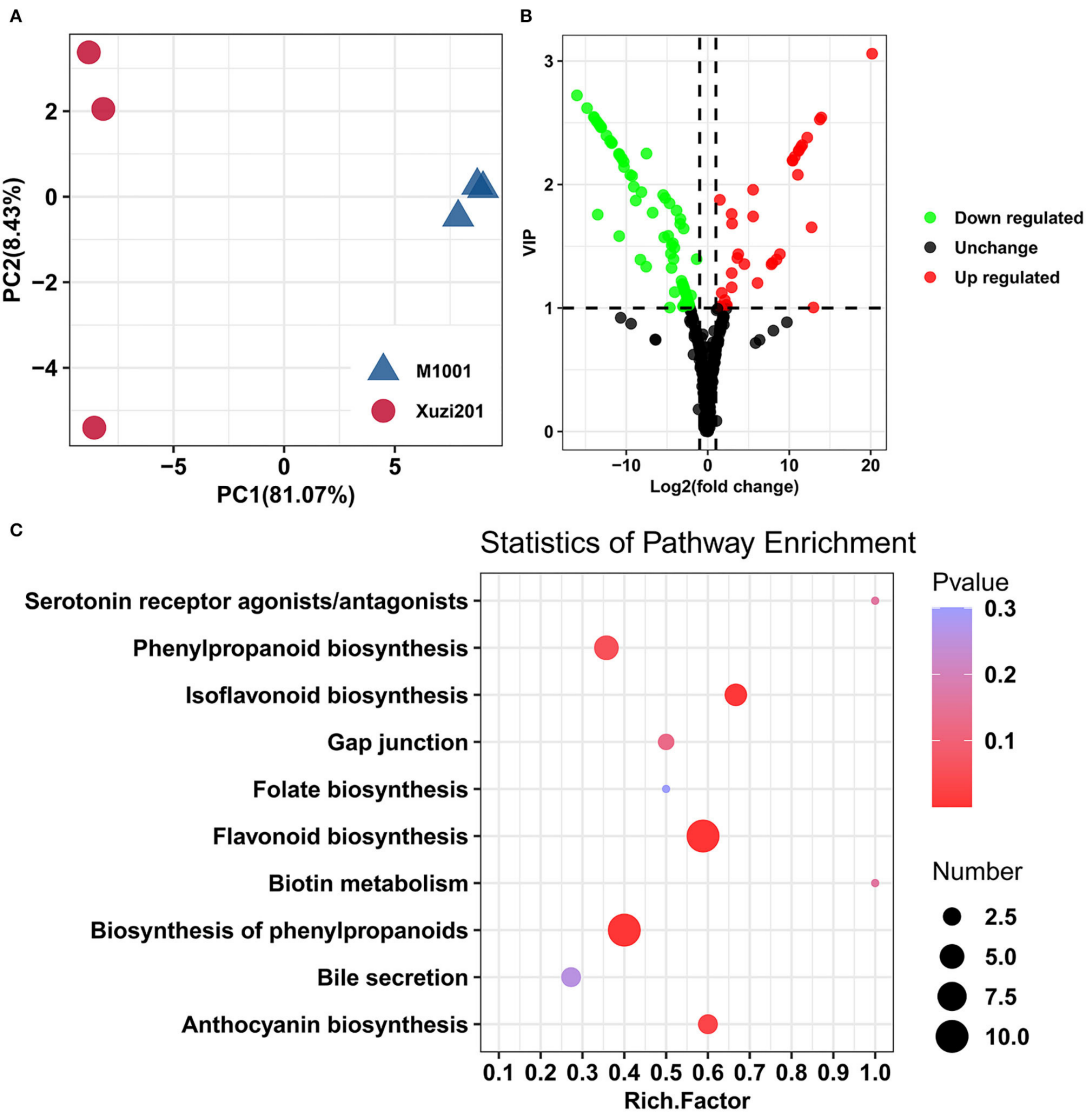
Metabolome analyses of tuberous roots of Xuzi201 and M1001. **(A)** PCA analyses of metabolites identified in Xuzi201 and M1001. X-axis represents the first principal component. Y-axis represents the second principal component. **(B)** Volcano plots displaying the upregulated, downregulated, and no regulated metabolites between Xuzi201 and M1001. Horizontal coordinates are Log_2_FC of metabolites, and vertical coordinates are variable important in projection (VIP) values of metabolites. The red, green, and black dots represent upregulated, downregulated, and unchanged metabolites. **(C)** Top ten KEGG enrichment terms of differentially expressed metabolites. The horizontal coordinate indicates the rich factor of each pathway, the vertical coordinate is the name of the pathway, and the color of the dot is the *P*-value; the redder it is, the more significant the enrichment. The size of the dots represents the number of differential metabolites enriched.

A systematic metabolic profiling of roots of two cultivars was carried out using a widely targeted metabolomics strategy. The Pearson's correlation coefficient among the three biological replicates of each cultivar was all >0.98, indicating a high repeatability of the generated metabolome data. Based on the PCA of the metabolites (Figure 2A), two cultivars were evidently separated. The first two principal components, namely, PC1 and PC2, took 81.07 and 8.43%, respectively. A total of 437 metabolites that were classified into 29 known classes were detected in the roots of these two sweet potato cultivars (Supplementary Table S3), among which, the most abundant metabolites were flavonoids, followed by organic acids, amino acids and their derivatives, and lipids.

The differentially accumulated metabolites (DAMs) screening results were presented in a volcano-shaped plot (Figure 2B). In total, 107 DAMs were detected between the two cultivars (Supplementary Table S4). In comparison with Xuzi201, M1001 had 35 upregulated and 72 downregulated metabolites. The top enriched KEGG terms among the DAMs were flavonoid biosynthesis, biosynthesis of phenylpropanoids, isoflavonoid biosynthesis, and anthocyanin biosynthesis (Figure 2C). Given the roles of flavonoids and anthocyanins in plant coloration, we deduced that the DAMs from the flavonoid biosynthesis and anthocyanin biosynthesis pathway are likely to be key metabolites underlying the color mutation of sweet potato flesh.

**Figure 3:**
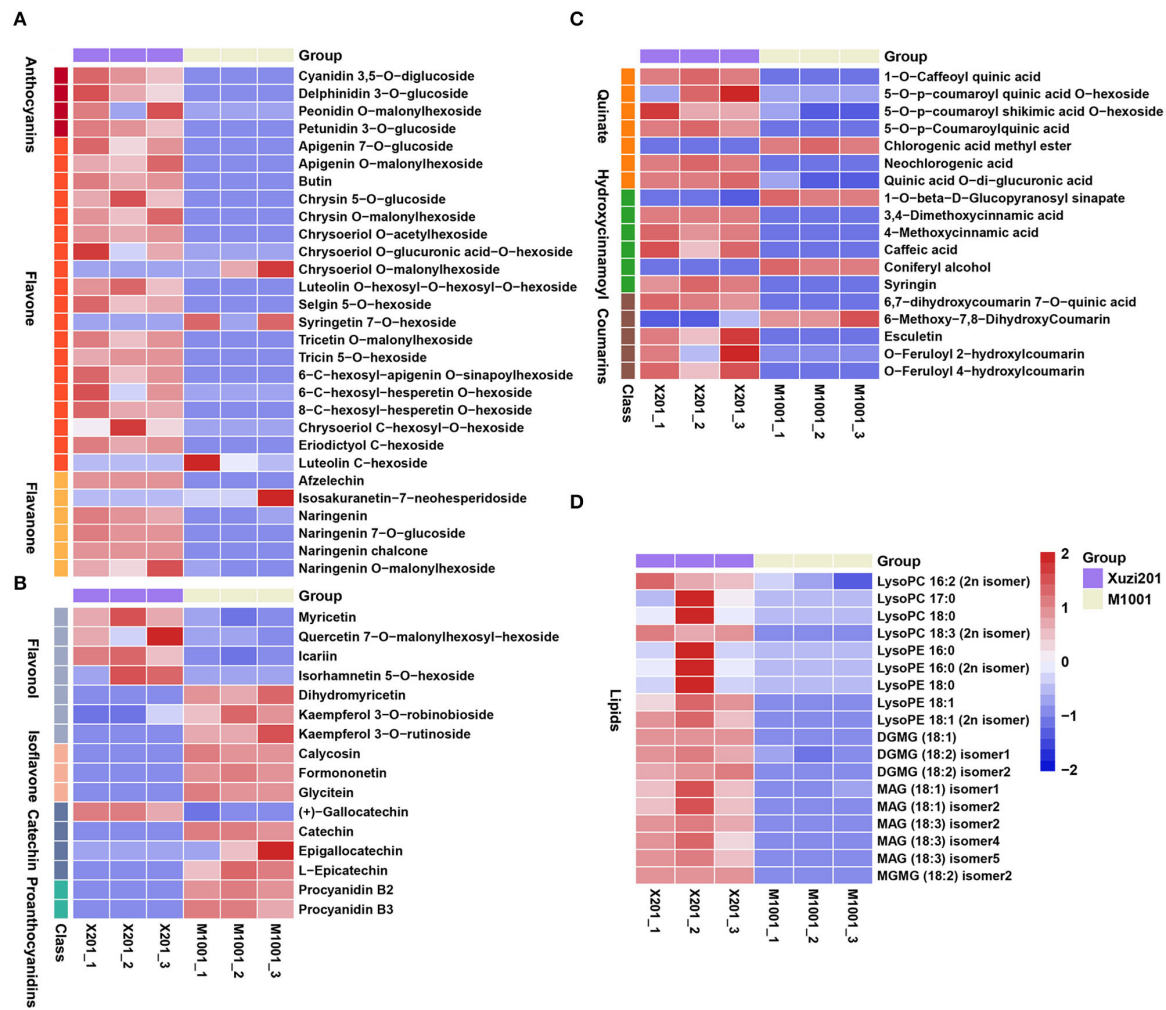
Heatmap of differentially accumulated metabolites (DAMs) in Xuzi201 and M1001. **(A)** anthocyanins, flavones, and flavanone; **(B)** flavonol, isoflavone, catechin, and proanthocyanidins; **(C)**: phenylpropanoid compounds; **(D)**: lipids. The content of each metabolite was scaled. Each example is visualized in a single column, and each metabolite is represented by a single row. Red indicates high abundance, whereas low relative metabolites are shown in blue.

Phenylalanine (Phe) is a product of the shikimic acid pathway, which converts simple precursors derived from carbohydrate metabolism into aromatic amino acids, and acts as an important precursor for flavonoids biosynthesis. In this study, Phe accumulated more in M1001 than Xuzi201 (Supplementary Table S4). Apart from Phe, 45 other DAMs in the flavonoid biosynthesis pathway were identified, including 4 anthocyanins, 19 flavones, 6 flavanones,7 flavonols, 3 isoflavones, 4 catechin derivatives, and 2 proanthocyanidins (Figures 3A,B). Seven quinate and its derivatives, 6 hydroxycinnamoyl derivatives, and 5 coumarins and their derivatives that were synthesized from the phenylpropanoid biosynthesis pathway were also identified as DAMs (Figure 3C). Anthocyanins are the most important flavonoid colorants in plants. Four types of anthocyanins were identified as DAMs in this study, including two cyanidin derivatives (cyanidin 3,5-O-diglucoside and peonidin O-malonylhexoside) and two delphinidin derivatives (delphinidin 3-O-glucoside and petunidin 3-O-glucoside). All of them were present in Xuzi201 and absent in M1001, which indicates that the missing of purple color in M1001 is probably due to the inhibition of anthocyanins biosynthesis. Except anthocyanins, almost all flavones and flavanones were downregulated in M1001, and among them, 15 flavones and 4 flavanones like apigenin, chrysin, and naringenin were identified only in Xuzi201, while no accumulation was found in M1001. Dihydromyricetin and kaempferol, two important flavonols, were abundantly accumulated in M1001 but not detected in Xuzi201. Other flavonols were downregulated in M1001. All isoflavones (calycosin, formononetin, and glycitein), catechins (except gallocatechin), and procyanidins (B2 and B3) were upregulated in M1001 and were not identified in Xuzi201 (Figures 3A,B). Besides, the DAMs synthesized from the phenylpropanoid biosynthesis pathway were nearly all downregulated except chlorogenic acid methyl ester, 1-O-beta-D-glucopyranosyl sinapate, coniferyl alcohol, and 6-methoxy-7,8-dihydroxycoumarin (Figure 3C). In addition, the accumulation of all identified lipids in DAMs was also reduced in M1001, including 9 glycerophospholipids and glycerolipids (Figure 3D).

### Volatile Metabolome Profiles of Purple and Cream Mutant of Sweet Potato

**Figure 4:**
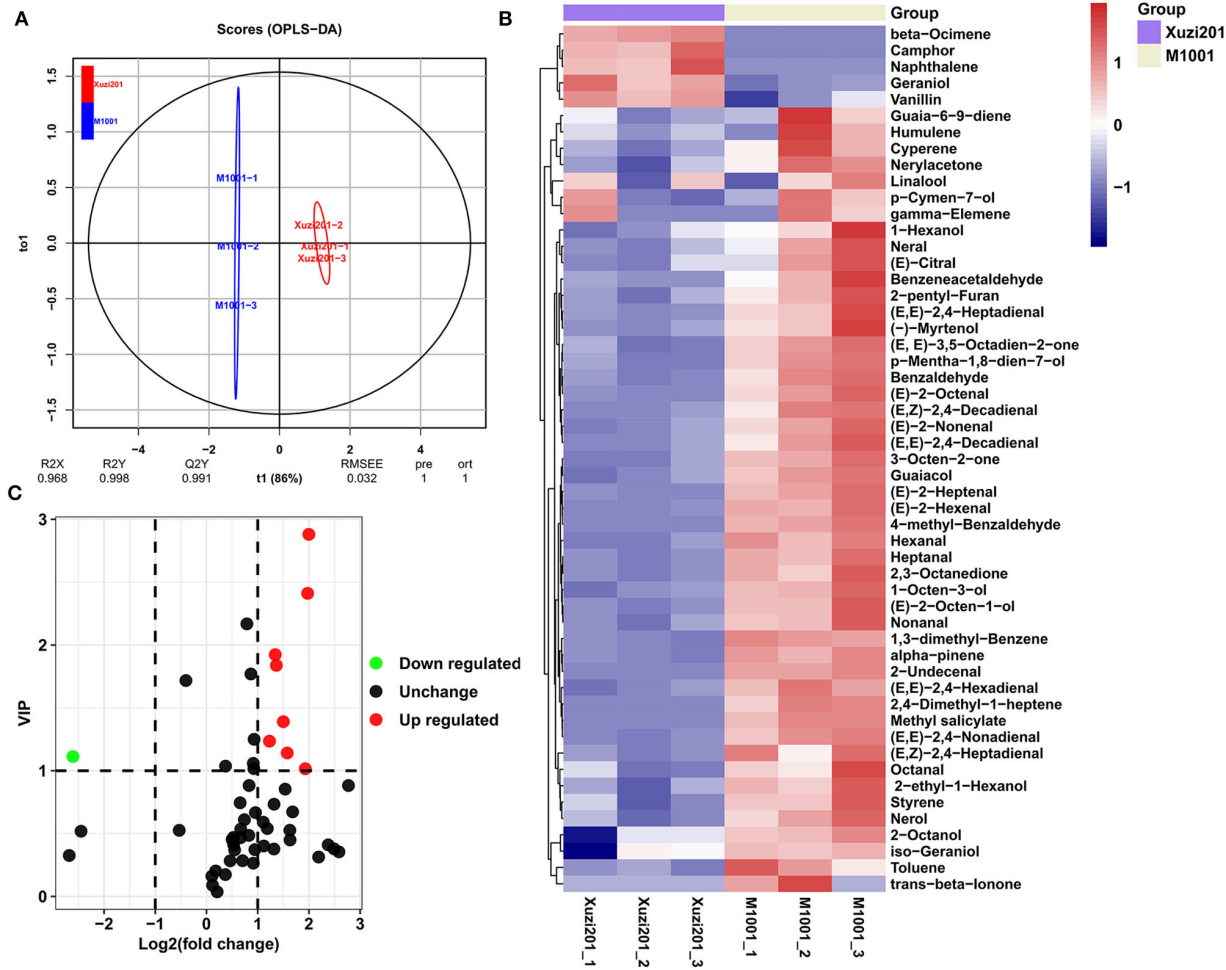
Volatile metabolites analyses of tuberous roots of Xuzi201 and M1001. **(A)** OPLS-DA model plots and loading plots for the Xuzi201 vs. M1001. **(B)** Heatmap visualization of the relative differences in volatile metabolites in Xuzi201 and M1001. The content of each metabolite was normalized to complete linkage hierarchical clustering. Each example is visualized in a single column, and each metabolite is represented by a single row. Red indicates high abundance, whereas low relative metabolites are shown in blue. **(C)** Volcano plots displaying the upregulated, downregulated, and no regulated volatile metabolites between Xuzi201 and M1001. Horizontal coordinates are Log_2_FC of metabolites, and vertical coordinates are variable important in projection (VIP) values of metabolites. The red, green, and black dots represent upregulated, downregulated, and unchanged metabolites.

Many phenylpropanoid compounds originating from Phe, such as benzeneacetaldehyde and benzaldehyde, providing honey and sweet odor, are important volatile compounds for sweet potato (Dudareva et al., 2013). In order to deeply understand the aromatic differences between these two cultivars, we profiled the volatiles from the tuberous roots of Xuzi201 and M1001 using HP-SPME GC–MS. A total of 53 volatile compounds were identified in the two cultivars. The detected volatile compounds included 21 aldehydes, 11 alcohols, 6 ketones, 9 alkenes, and 6 other compounds (Supplementary Table S5). Orthogonal projections to latent structures discriminant analysis (OPLS-DA) was performed on all samples. The OPLS-DA results (Figure 4A) showed that the value of Q^2^Y was 0.991, indicating the reliability of the volatile profile, and the two cultivars were evidently separated, which indicated significant aromatic differences between them.

A cluster heatmap was made based on the relative amount of all the volatiles (Figure 4B). Compared with Xuzi201, only five compounds were reduced in M1001, while most of the volatile compounds increased. The differentially accumulated volatile compounds between the two cultivars were determined based on the VIP≥1 and Log_2_FC ≥1 or Log_2_FC ≤-1. It could be seen from the volcano-shaped plot that the relative contents of 9 volatiles showed a significant difference and the other 44 volatiles had no clear change between these two cultivars. Among the 9 differentially accumulated volatiles, 8 compounds were upregulated, while the camphor, a monoterpenoid with a strong mothball-like odor, was the only compound downregulated in M1001 (Figure 4C). Among the 8 upregulated volatiles in M1001, one was guaiacol, a common volatile presenting smoke odor in sweet potato. The other seven volatiles [(E,E)-2,4-decadienal, (E,Z)-2,4-decadienal, (E)-2-heptenal, (E)-2-octenal, (E)-2-nonenal, hexanal, (E,E)-2,4-nonadienal] were produced from the linolenic acid degradation. These results indicated that the coloration of purple flesh color had no significant effect on the aromatic compounds derived from phenylalanine in sweet potato.

### Transcriptome Profiling of Purple and Cream Mutant of Sweet Potato

To understand the molecular basis of the metabolic differences between Xuzi201 and its mutant M1001, transcriptome sequencing of the roots of these two cultivars was carried out. A total of 50 G clean base was obtained, with the number of 7.65–8.8 G clean base achieved for each sample after filtration. The average Q30 for Xuzi201 and M1001 was 92.8 and 92.77%, respectively. After assembling the clean reads, a total of 101,536 unigenes were obtained with a maximum length of 16,845 bp, an average length of 524 bp, and an average GC% ratio of 42.72%. Overall, the data indicated that the sequencing was of high quality and met the requirements for further analysis. Among them, 42,303, 19,060, 34,163, 32,416, 43,336, and 60,277 unigenes corresponded to GO, KEGG, KOG, Pfam, Swissport, and NR database, respectively, and totally, 61,093 unigenes were annotated (Supplementary Table S6). Pearson correlation coefficients were calculated between samples based on the number of FPKM values. The correlation coefficients between the biological replicates of the same genotype were >0.95, indicating good reproducibility of the biological repeats. These results suggested that the quantity and quality of the sequencing data were suitable for the downstream analysis.

**Figure 5:**
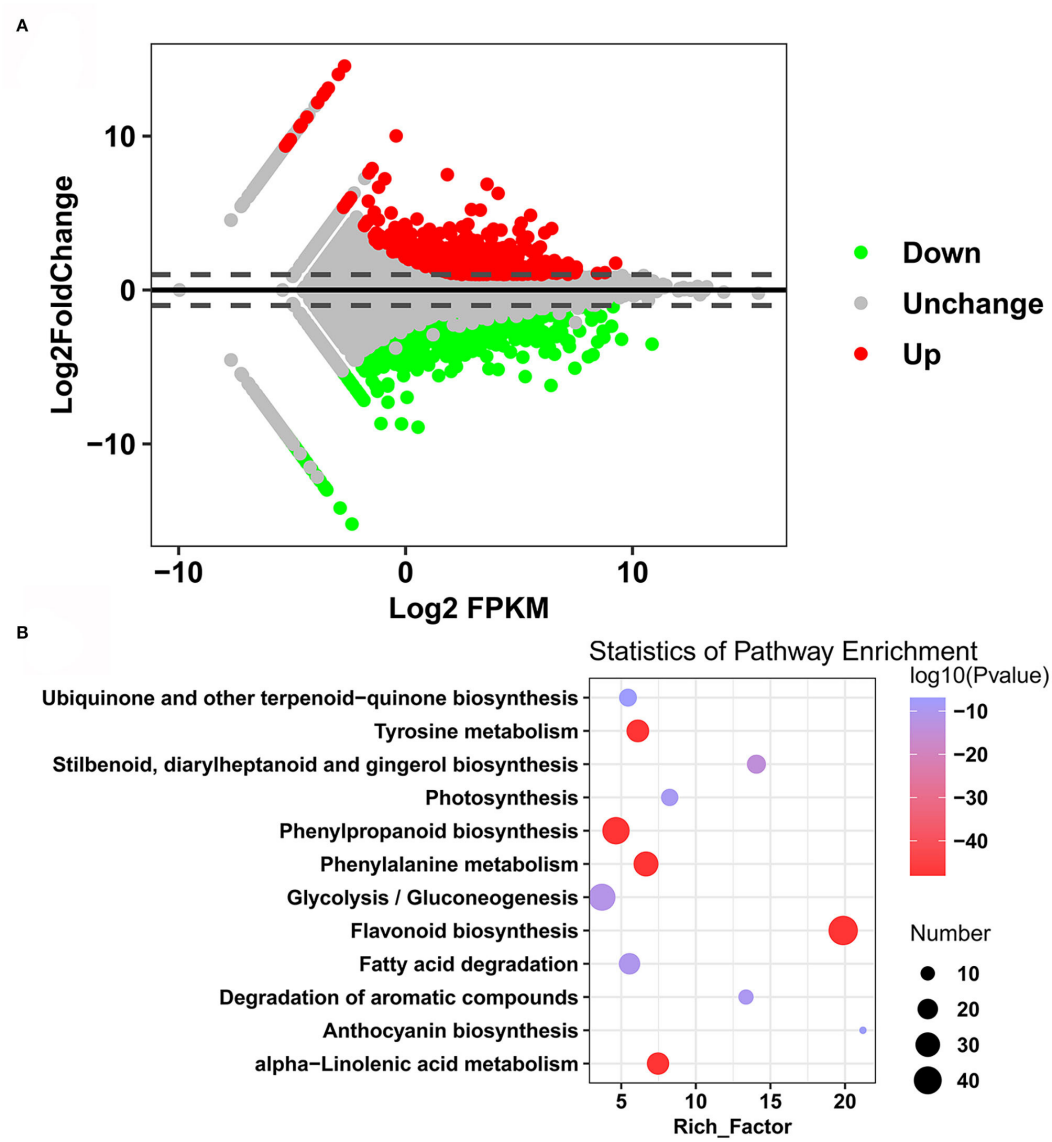
DEGs and KEGG enrichment analyses of roots of Xuzi201 and M1001. **(A)** M-vs.-A plot (MA) displaying the upregulated, downregulated, and unchanged expression genes between Xuzi201 and M1001. Horizontal coordinates are Log_2_FPKM of expression genes, and vertical coordinates are Log_2_ fold change of expression genes. The red, green, and black dots represent the genes expression upregulated, downregulated, and unchanged. **(B)** Top 12 KEGG enrichment terms of differentially expressed genes. The horizontal coordinate indicates the rich factor of each pathway, the vertical coordinate is the name of the pathway, and the color of the dot is the *P*-value; the redder it is, the more significant the enrichment. The size of the dots represents the number of differential metabolites enriched.

The differentially expressed genes (DEGs) between the two sweet potato cultivars were analyzed with DESeq2 and determined based on the FDR <0.01 and Log_2_FC ≥1 or Log_2_FC ≤-1. A total of 1,593 DEGs were identified between Xuzi201 and M1001: 554 upregulated genes and 1,039 downregulated genes in M1001 (Figure 5A, Supplementary Table S7). To analyze the functions of the DEGs between Xuzi201 and M1001, a GO analysis was performed using GOseq. GO enrichment showed that the DEGs were enriched in the terms “oxidation reduction process,” “anthocyanin-containing compounds biosynthetic process,” “oxidoreductase activity,” “alcohol dehydrogenase (NAD) activity,” and “trans-cinnamate 4-monooxygenase activity” (Supplementary Figure S1). Then, KEGG analysis was performed to examine the DEGs-associated pathways. The top 12 enriched pathways were shown in Figure 5B. “Flavonoid biosynthesis,” “Phe metabolism,” “phenylpropanoid biosynthesis,” and “anthocyanin biosynthesis” were intensively enriched. Altogether, the GO and KEGG analysis results indicated that the DEGs enriched significantly in phenylpropanoid biosynthesis, flavonoid biosynthesis, and anthocyanin biosynthesis pathway.

### Association Metabolic and Transcriptomic Analysis of Purple and Cream Mutant of Sweet Potato

Both metabolic analysis and transcriptomic analysis results highlighted that the flavonoid and anthocyanin biosynthesis and related upstream pathways were enriched. In order to further analyze the relationship between those enriched DAMs and DEGs, an association analysis between metabolome and transcriptome was performed. The flavonoid biosynthesis pathway was the most significantly enriched pathway (Supplementary Figure S2). Then, the correlation coefficients between DAMs and DEGs were calculated, and the correlations with the coefficient of *R*^2^ > 0.8 were selected as significant. In total, 300 significant correlations between 93 transcripts and 35 metabolites were identified, each metabolite correlated with one or more transcripts. Among 35 metabolites, 3 anthocyanins and 20 flavones and flavanols were included (Supplementary Table S8). Two flavones, selgin 5-O-hexoside and 8-C-hexosyl-hesperetin O-hexoside, correlated with the highest number of transcripts: 34 and 33 transcripts, respectively. Similarly, each transcript also correlated with one or several metabolites.

**Figure 6:**
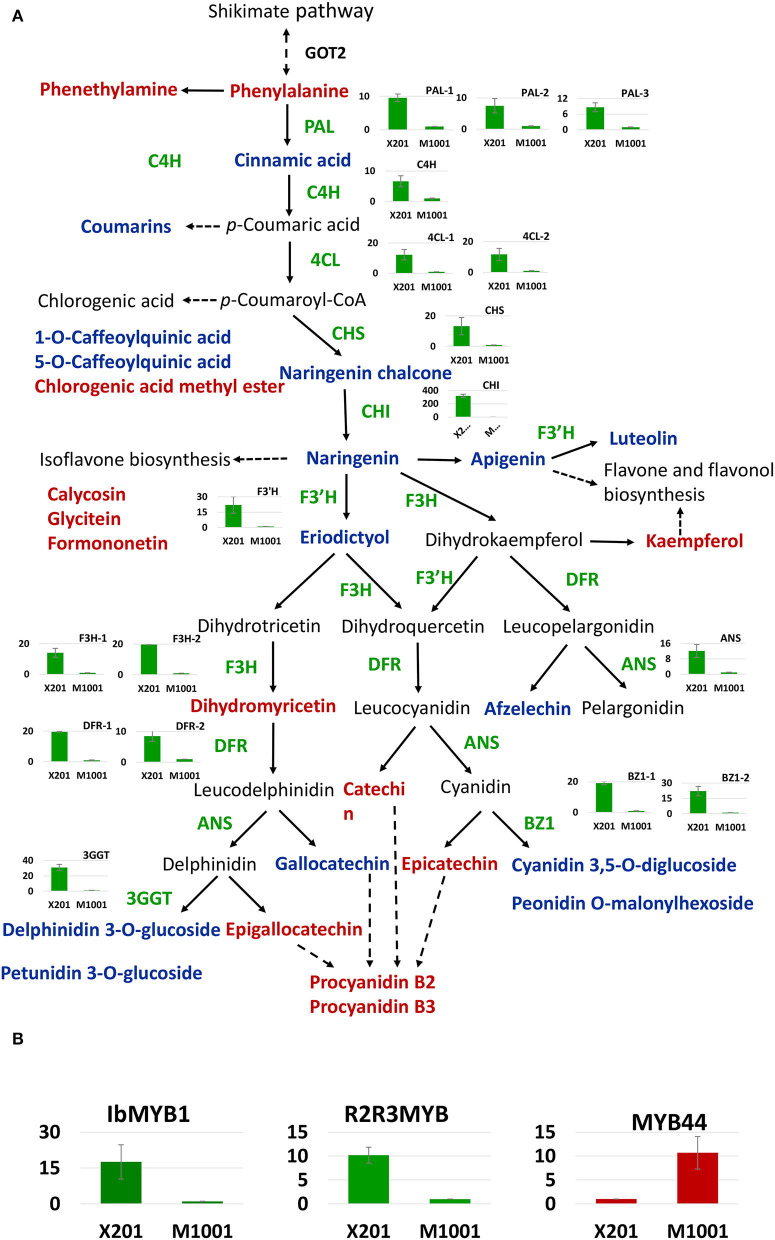
Proposed anthocyanin biosynthesis pathway in sweet potato. X201 and M1001 presented Xuzi201 and M1001, respectively. **(A)** Transformation of differentially accumulated metabolites (DAMs) and differentially expressed genes (DEGs) in phenylpropanoid metabolism pathway and flavonoid biosynthesis in purple sweet potato and its mutant. Metabolites in red and blue indicate up-accumulated and down-accumulated in M1001, respectively. The histogram presents the expression level of corresponding structural genes in two varieties, green bar means downregulated in M1001. Enzymes in this pathway are shown as follows: GOT2, aspartate aminotransferase; PAL, phenylalanine ammonia lyase; C4H, cinnamate 4-hydroxylase; 4CL, 4-coumarateCoA ligase; CHS, chalcone synthase; CHI, chalcone isomerase; F3H, flavanone 3-hydroxylase; F3′H, flavonoid 3′-hydroxylase; DFR, dihydroflavonol 4-reductase; ANS, anthocyanidin synthase; BZ1: anthocyanidin 3-O-glucosyltransferase gene; 3GGT: anthocyanidin 3-O-glucoside 2”-O-glucosyltransferase. **(B)** Differentially expressed transcript factors involved in the pathway. The histogram presents the expression level of corresponding transcript factors in two varieties, red and green bar means up and downregulated in M1001, respectively. All the genes expression data presented in the figure were from qRT-PCR expression analysis.

The 93 transcripts were annotated, and the flavonoid and anthocyanin biosynthesis and regulation-related transcripts were selected. Totally, 49 differentially expressed genes and TFs were identified (Supplementary Table S9), containing most of the key enzyme-encoding genes in the flavonoid and anthocyanin biosynthesis pathway. Based on the changes in the content of DAMs and DEGs identified in our study, the colorless formation mechanism in the mutant was analyzed (Figure 6A). In the mutant, due to the expression of phenylalanine lyases (*PAL*s) was substantially reduced, the anthocyanin biosynthesis precursor Phe was more accumulated, and cinnamic acid was downregulated. Subsequently, a series of structural genes in the flavonoids containing cinnamate 4-hydroxylase gene (*C4H*), 4-coumarate CoA ligase gene (*4CL*), chalcone synthase gene (*CHS*), chalcone isomerase gene (*CHI*), and flavanone 3′-hydroxylase gene (*F3*′*H*) presented significant low expression level in M1001. Thus, some important intermediates like naringenin chalcone, naringeni,n and eriodictyol, which were produced by these structural genes-encoded enzymes, were only accumulated in Xuzi201 and were not identified in M1001. Finally, all genes related to anthocyanins biosynthesis, such as flavanone 3-hydroxylase gene (*F3H*), dihydroflavonol 4-reductase gene (*DFR*), anthocyanidin synthase gene (*ANS*), anthocyanidin 3-O-glucosyltransferase gene (*BZ1*), and anthocyanidin 3-O-glucoside 2”-O-glucosyltransferase (*3GGT*), showed significant downregulation in M1001, and thus, anthocyanins cannot be accumulated, which formed the cream flesh. But the colorless metabolites catechins and proanthocyanidins were synthesized from the same precursor (Phe). In this study, three catechins and two proanthocyanidins were indeed more enriched in M1001.

Moreover, certain TFs, such as *bHLH2, MYB1, MYB44*, and *R2R3 MYB*(AB576766.1), related to the biosynthesis of flavonoid and anthocyanin, were identified in this study. The expression levels of *MYB1, MYB44*, and *R2R3 MYB* were verified by RT-qPCR (Figure 6B). The TFs were almost all downregulated in M1001 except *MYB44*, which is a repressor of anthocyanin in sweet potato. Otherwise, the aspartate aminotransferase (*GOT2*) gene, which is related to Phe biosynthesis, was upregulated in M1001, and two genes [glutathione *S*-transferase (*GST*) and multidrug and toxic compound extrusion (*MATE*)] related to anthocyanins transportation from the endoplasmic reticulum to vacuole were downregulated. The expression of these DEGs was consistent with the metabolite accumulation pattern in sweet potato root.

**Figure 7:**
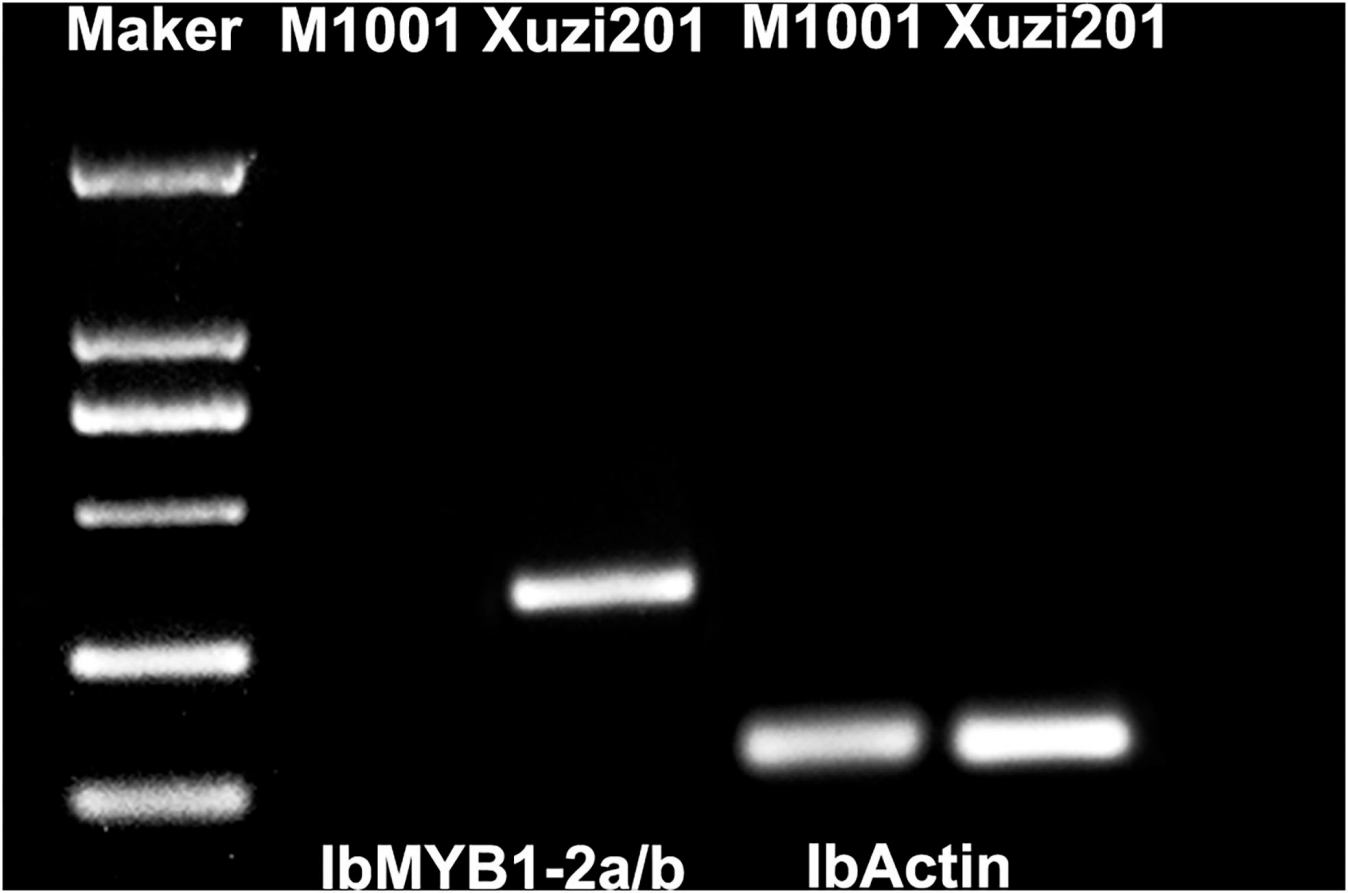
Genotype of IbMYB1-2a/b in Xuzi201 and M1001. X201 and M1001 presented Xuzi201 and M1001, respectively.

In view of the *IbMYB1* gene has been reported as a mutate location in some purple sweet potato and responsible for anthocyanin accumulation in the storage roots (Tanaka et al., 2012; Zhang D. et al., 2021), we amplified the gene *IbMYB1-2a/b* with the specific makers. The result showed that IbMYB1-2a/b was only amplified in Xuzi201 and lost in M1001 (Figure 7). This result indicated that the M1001 has a mutation in IbMYB1 gene.

### Verification of RNA Sequencing Results by qRT-PCR

To verify the accuracy of the transcriptome data from the RNA sequencing analysis, 20 unigenes (17 structural genes and 3 TFs) involved in the coloration and quality of sweet potato roots were selected for qRT-PCR expression analysis. The genes expression level results were presented as a histogram in Figure 6. The results showed that the RNA-seq and qRT-PCR data were positively correlated (Supplementary Figure S3), and the genes' expression level of qRT-PCR was consistent with the FPKM value of RNA-seq results (*R*^2^ = 0.78). Overall, our results indicated that the RNA sequencing data were valid and reliable in the study.

## Discussion

As sweet potato is recognized as a healthy food, the consumers' demand has been increasing in recent years, especially those of PFSPs, which contain high levels of anthocyanins (Wang et al., 2018a). In this study, a cream flesh mutant M1001 was obtained through ^60^Co γ-ray radiation of a PFSP cultivar Xuzi201. The phenotype between Xuzi201 and M1001 is highly consistent, except for root skin and flesh color. Thus, we used them as plant materials to explore the potential regulatory mechanism responsible for anthocyanins accumulation and the effect of different flesh colors on the quality of sweet potato storage roots through an integrated metabolome and transcriptome analysis.

Preliminary quality analyses were carried out for the storage roots of these two varieties. The results showed that the flavonoids and total anthocyanin content of the cream mutant were all lower than Xuzi201. This result is consistent with wide-targets metabolome analyses. In total, 106 flavonoids including 8 anthocyanins were identified in these two cultivars; among them, 86 flavonoids containing 8 anthocyanins were identified in Xuzi201, and 70 flavonoids and 4 anthocyanins were identified in M1001, and the relative content of flavonoids in Xuzi201 was about 100 times than that in M1001. Meanwhile, we noticed that 4 anthocyanins (i.e., cyanidin 3,5-O-diglucoside, delphinidin 3-O-glucoside, petunidin 3-O-glucoside, and peonidin O-malonylhexoside) were absent in M1001, and the other 4 anthocyanins (i.e., Malvidin 3-O-galactoside, Malvidin 3-O-glucoside, Cyanidin, and Peonidin) detected in all samples had no significant differences between these two varieties. Thus, these 4 anthocyanins absent in M1001 were speculated as the key anthocyanin derivatives responsible for the pigmentation of Xuzi201, and the other 4 anthocyanins were not the main colorant pigments in sweet potato. This result is consistent with previous reports that more than 30 individual anthocyanins have been identified in different PFSPs, and 3,5-diglucoside derivatives of cyanidin or derivatives of peonidin are the dominant pigments (Kim et al., 2012).

Anthocyanins, the final production of the flavonoid biosynthesis pathway, were strongly associated with the composition and the content of its upstream flavonoids. Phe is an important precursor for flavonoids biosynthesis, and it was synthesized from shikimate pathway, and tryptophan and tyrosine were also produced from this pathway. In this study, Phe and its derivative phenethylamine (phe-phe), and tryptophan's derivative acetyl tryptophan were more enriched in M1001. In addition, the transcriptome analysis result showed that the expression of *GOT2* gene, which is involved in the biosynthesis and metabolism of Phe, was significantly higher in M1001 than in Xuzi201. It has been reported that the Phe biosynthesis-related enzyme is feedback regulated by Phe in Arabidopsis and rice (Yamada et al., 2008; Huang et al., 2010). Therefore, we suspected that the higher amount of Phe in M1001 did not flow in the downstream flavonoid synthesis pathway but promoted the production of phe-phe and acetyl tryptophan in a feedback regulation way. The flavonoid biosynthesis in M1001 was inhibited from the starting point Phe, and then, the downstream flavonoids in the biosynthesis pathway of anthocyanins were all downregulated. However, the amount of Phe in Xuzi201 flowed to anthocyanin biosynthesis and produced abundant intermediate flavonoids and anthocyanins.

A previous study has proven that the genes involved in the anthocyanin accumulation are categorized into two parts, namely, structural genes, and regulatory genes (Wang et al., 2021). The structural genes include the early biosynthesis genes (*PAL, C4H, 4CL, CHS, CHI, F3H*) and anthocyanin-specific biosynthesis genes (*F3*′*H, F3*′*5*′*H, DFR, ANS, BZ1*, and *3GGT*) (Jaakola et al., 2002). In our study, except *F3*′*5*′*H*, the other structural genes were identified, and all of them were downregulated in M1001. The low transcriptional level of structural genes led to the decreased content of flavonoids and anthocyanins. Nevertheless, considering that the structural genes could be expressed in a low level and synthesized some anthocyanins in M1001, we postulate that the structural genes were not the primary cause of the flesh color mutant, and the low expression level of them was caused by the regulation of TFs. Four important TFs (i.e., *bHLH2, R2R3-MYB, MYB1*, and *MYB44*) were identified as DEGs. *MYB1*, a member of *R2R3-MYB* TF, previously reported as specifically expressed in sweet potato roots, has been identified as an activator for the promoters of a number of anthocyanin pathway genes in several crops including sweet potato (Mano et al., 2007; Park et al., 2015). bHLH TFs, the partner of MYBs, can interact with MYBs to regulate anthocyanin biosynthesis (Liu et al., 2016). In this study, the expression levels of *bHLH2, R2R3-MYB*, and *MYB1* were significantly lower in M1001 than in Xuzi201. Moreover, *MYB44*, an anthocyanin biosynthesis repressor, was upregulated in M1001. We hypothesize that the differential regulation of *bHLH2, R2R3-MYB, MYB1*, and *MYB44* might be responsible for the lack of anthocyanins in M1001. Since the previous study showed that the absence of the *IbMYB1-2* sequences in the white flesh mutant of purple sweet potato caused a lack of anthocyanin accumulation in the storage roots (Tanaka et al., 2012), we detected this gene in Xuzi201 and M1001 and confirmed that it was absent in M1001. Thus, the absence of IbMYB1-2a/b was an important reason for the lack of anthocyanins in M1001.

In addition to the anthocyanin synthesis-related genes, a few genes involved in anthocyanin transport were identified. Anthocyanin pigments are synthesized at the cytoplasmic surface of the endoplasmic reticulum and are finally transported into the vacuole. GSTs attach glutathione to individual anthocyanin, which tags them for MATE transporters at tonoplast (Alfenito et al., 1998; Edwards et al., 2000). Both *GSTs* and *MATE* were downregulated in M1001. Meanwhile, the genes encoding a 24 kDa vacuolar protein VP24 were downregulated, and this protein was involved in vacuolar transport and/or accumulation of anthocyanin synthesized in the cytosol (Xu et al., 2001). This result reveals that as anthocyanin biosynthesis was reduced, the proteins corresponding to anthocyanin pigments transport to and storage in vacuole were also downregulated.

Furthermore, we would like to evaluate whether there is a correlation between flesh coloration and quality attributes in sweet potato. Considering that Phe was the origin for phenylpropanoid compounds, which is the second largest class of plant volatile compounds (Dudareva et al., 2013), the volatile compounds of two varieties were detected, and no differentially accumulated volatile compounds synthesized from Phe were identified. Nevertheless, the volatile compounds [(E,E)-2,4-decadienal, (E,Z)-2,4-decadienal, (E)-2-heptenal, (E)-2-octenal, (E)-2-nonenal, hexanal, (E,E)-2,4-nonadienal] produced from linolenic acid degradation had more accumulation in M1001. The lipids in M1001 had a relatively low concentration; these lipids probably formed linolenic acid, and the decreased amount of lipids finally produced (E,E)-2,4-decadienal, (E,Z)-2,4-decadienal, (E)-2-heptenal, (E)-2-octenal, (E)-2-nonenal, hexanal, and (E,E)-2,4-nonadienal. However, the connection between lipids and these volatile compounds is still unclear, and it will be a starting point for further study. Moreover, the starch content was a significant difference in M1001 and Xuzi201. The expression level of genes related to starch biosynthesis and metabolic rate were checked, and no DEGs were identified. Thus, we considered that differences in starch content are caused by differences in protein levels, and we will verify it in further study.

In this study, the colorless formation mechanism and chemical composition were revealed through a combination analysis of metabolome and transcriptome using the PFSP and its cream fleshed mutant as plant materials. What's more, the quality differences caused by flesh color mutation were also analyzed. These results not only enable us to identify genes for targeted genetic engineering to improve the nutritional value of sweet potato but also highlight the potential usage of sweet potato as a functional food and provide direction for the breeding of high-quality sweet potato.

## Data Availability Statement

The datasets presented in this study can be found in online repositories. The names of the repository/repositories and accession number(s) can be found at: https://ngdc.cncb.ac.cn/?lang=en, PRJCA008065.

## Author Contributions

RZ and ZW: conceptualization and methodology. ML: material provider. RZ and XM: formal analysis. RZ: data curation, writing original draft, and visualization. ZW, CT, BJ, and ZY: writing review and editing. ZW: funding acquisition. All authors have read and agreed to the published version of the manuscript.

## Funding

This work was supported by grants from the China Agriculture Research System of MOF and MARA, the National Key R&D Program of China (nos. 2019YFD1000700 and 2019YFD1000701), the Guangdong Modern Agro-industry Technology Research System (2021KJ111), and the Presidential Foundation of Guangdong Academy of Agricultural Sciences (BZ202009).

## Conflict of Interest

The authors declare that the research was conducted in the absence of any commercial or financial relationships that could be construed as a potential conflict of interest.

## Publisher's Note

All claims expressed in this article are solely those of the authors and do not necessarily represent those of their affiliated organizations, or those of the publisher, the editors and the reviewers. Any product that may be evaluated in this article, or claim that may be made by its manufacturer, is not guaranteed or endorsed by the publisher.

## References

[B1] Aharoni A. De Vos C. H. R. Wein M. Sun Z. Greco R. Kroon A.. (2001). The strawberry FaMYB1 transcription factor suppresses anthocyanin and flavonol accumulation in transgenic tobacco. Plant J. 28, 319–332. 10.1046/j.1365-313X.2001.01154.x 11722774

[B2] Alam M. K. (2021). A comprehensive review of sweet potato (Ipomoea batatas [L.] Lam): revisiting the associated health benefits. Trends Food Sci. Technol. 115, 512–529. 10.1016/j.tifs.2021.07.001

[B3] Alfenito M. R. Souer E. Goodman C. D. Buell R. Mol J. Koes R.. (1998). Functional complementation of anthocyanin sequestration in the vacuole by widely divergent glutathione S-transferases. Plant Cell. 10, 1135–1149. 10.1105/tpc.10.7.1135 9668133 PMC144053

[B4] Asadi K. Ferguson L. R. Philpott M. Karunasinghe N. (2017). Cancer-preventive properties of an anthocyanin-enriched sweet potato in the APC^MIN^ mouse model. J. Cancer Prev. 22, 135–146. 10.15430/JCP.2017.22.3.135 29018778 PMC5624454

[B5] Bovell Benjamin A. C. (2007). Sweet potato: a review of its past, present, and future role in human nutrition. Adv. Food Nutr. Res. 52, 1–59. 10.1016/S1043-4526(06)52001-7 17425943

[B6] Chen W. Gong L. Guo Z. Wang W. Zhang H. Liu X.. (2013). A novel integrated method for large-scale detection, identification, and quantification of widely targeted metabolites: application in the study of rice metabolomics. Mol. Plant. 6, 1769–1780. 10.1093/mp/sst080 23702596

[B7] de Albuquerque T. M. R. Sampaio K. B. de Souza E. L. (2019). Sweet potato roots: unrevealing an old food as a source of health promoting bioactive compounds – a review. Trends Food Sci. Technol. 85, 277–286. 10.1016/j.tifs.2018.11.006

[B8] Dudareva N. Klempien A. Muhlemann J. K. Kaplan I. (2013). Biosynthesis, function and metabolic engineering of plant volatile organic compounds. New Phytol. 198, 16–32. 10.1111/nph.12145 23383981

[B9] Edwards R. Dixon D. P. Walbot V. (2000). Plant glutathione S-transferases: enzymes with multiple functions in sickness and in health. Trends Plant Sci. 5, 193–198. 10.1016/S1360-1385(00)01601-0 10785664

[B10] FAOSTAT (2021). Available online at: http://www.fao.org/faostat/zh/#data/QC (accessed November 23, 2021).

[B11] Guo J. Zhou W. Lu Z. Li H. Li H. Gao F. (2015). Isolation and functional analysis of chalcone isomerase gene from purple-fleshed sweet potato. Plant Mol. Biol. Rep. 33, 1451–1463. 10.1007/s11105-014-0842-x

[B12] Hu M. Lu Z. Guo J. Luo Y. Li H. Li L.. (2016). Cloning and characterization of the cDNA and promoter of UDP-glucose:flavonoid 3-O-glucosyltransferase gene from a purple-fleshed sweet potato. S. Afr. J. Bot. 106, 211–220. 10.1016/j.sajb.2016.07.018

[B13] Huang T. Tohge T. Lytovchenko A. Fernie A. R. Jander G. (2010). Pleiotropic physiological consequences of feedback-insensitive phenylalanine biosynthesis in *Arabidopsis thaliana*. Plant J. 63, 823–835. 10.1111/j.1365-313X.2010.04287.x 20598094

[B14] Jaakola L. Määttä K. Pirttilä A. M. Tärränen R. Kärenlampi S. Hohtola A. (2002). Expression of genes involved in anthocyanin biosynthesis in relation to anthocyanin, proanthocyanidin, and flavonol levels during bilberry fruit development. Plant Physiol. 130, 729–739. 10.1104/pp.006957 12376640 PMC166602

[B15] Jang H.-H. Kim H.-W. Kim S.-Y. Kim S.-M. Kim J.-B. Lee Y.-M. (2019). *In vitro* and *in vivo* hypoglycemic effects of cyanidin 3-caffeoyl-p-hydroxybenzoylsophoroside-5-glucoside, an anthocyanin isolated from purple-fleshed sweet potato. Food Chem. 272, 688–693. 10.1016/j.foodchem.2018.08.010 30309599

[B16] Kim C. Y. Ahn Y. O. Kim S. H. Kim Y.-H. Lee H.-S. Catanach A. S.. (2010). The sweet potato IbMYB1 gene as a potential visible marker for sweet potato intragenic vector system. Physiol. Plant. 139, 229–240. 10.1111/j.1399-3054.2010.01365.x 20163556

[B17] Kim H. J. Koo K. A. Park W. S. Kang D. M. Kim H. S. Lee B. Y.. (2020). Anti-obesity activity of anthocyanin and carotenoid extracts from color-fleshed sweet potatoes. J. Food Biochem. 44, e13438. 10.1111/jfbc.13438 32812262

[B18] Kim H. W. Kim J. B. Cho S. M. Chung M. N. Lee Y. M. Chu S. M.. (2012). Anthocyanin changes in the Korean purple-fleshed sweet potato, Shinzami, as affected by steaming and baking. Food Chem. 130, 966–972. 10.1016/j.foodchem.2011.08.031

[B19] Leksrisompong P. P. Whitson M. E. Truong V. D. Drake M. A. (2012). Sensory attributes and consumer acceptance of sweet potato cultivars with varying flesh colors. J. Sens. Stud. 27, 59–69. 10.1111/j.1745-459X.2011.00367.x

[B20] Lepiniec L. Debeaujon I. Routaboul J.-M. Baudry A. Pourcel L. Nesi N.. (2006). Genetics and biochemistry of seed flavonoids. Annu. Rev. Plant Biol. 57, 405–430. 10.1146/annurev.arplant.57.032905.105252 16669768

[B21] Li G. Lin Z. Zhang H. Liu Z. Xu Y. Xu G.. (2019). Anthocyanin accumulation in the leaves of the purple sweet potato (*Ipomoea batatas* L.) cultivars. Molecules 24, 3743. 10.3390/molecules24203743 31627373 PMC6832942

[B22] Liu X. Xiang M. Fan Y. Yang C. Zeng L. Zhang Q.. (2017). A root-preferential DFR-like gene encoding dihydrokaempferol reductase involved in anthocyanin biosynthesis of purple-fleshed sweet potato. Front. Plant Sci. 8, 279. 10.3389/fpls.2017.00279 28293252 PMC5329058

[B23] Liu Y. Kui L. W. Espley R. V. Li W. Yang H. Yu B.. (2016). Functional diversification of the potato R2R3 MYB anthocyanin activators AN1, MYBA1, and MYB113 and their interaction with basic helix-loop-helix cofactors. J. Exp. Bot. 67, 2159–2176. 10.1093/jxb/erw014 26884602 PMC4809278

[B24] Love M. I. Huber W. Anders S. (2014). Moderated estimation of fold change and dispersion for RNA-seq data with DESeq2. Genome Biol. 15, 1–21. 10.1186/s13059-014-0550-8 25516281 PMC4302049

[B25] Mano H. Ogasawara F. Sato K. Higo H. Minobe Y. (2007). Isolation of a regulatory gene of anthocyanin biosynthesis in tuberous roots of purple-fleshed sweet potato. Plant Physiol. 143, 1252–1268. 10.1104/pp.106.094425 17208956 PMC1820918

[B26] Montilla E. C. Hillebrand S. Butschbach D. Baldermann S. Watanabe N. Winterhalter P. (2010). Preparative isolation of anthocyanins from Japanese purple sweet potato (*Ipomoea batatas L*.) varieties by high-speed countercurrent chromatography. J. Agric. Food Chem. 58, 9899–9904. 10.1021/jf101898j 20731350

[B27] Park S. C. Kim Y. H. Kim S. H. Jeong Y. J. Kim C. Y. Lee J. S.. (2015). Overexpression of the IbMYB1 gene in an orange-fleshed sweet potato cultivar produces a dual-pigmented transgenic sweet potato with improved antioxidant activity. Physiol. Plant. 153, 525–537. 10.1111/ppl.12281 25220246

[B28] Pfaffl M. W. (2001). A new mathematical model for relative quantification in real-time RT–PCR. Nucleic Acids Res. 29, 45. 10.1093/nar/29.9.e45 11328886 PMC55695

[B29] Qiu W. Su W. Cai Z. Dong L. Li C. Xin M.. (2020). Combined analysis of transcriptome and metabolome reveals the potential mechanism of coloration and fruit quality in yellow and purple *Passiflora edulis* Sims. J. Agric. Food Chem. 68, 12096–12106. 10.1021/acs.jafc.0c03619 32936632

[B30] Shekhar S. Mishra D. Buragohain A. K. Chakraborty S. Chakraborty N. (2015). Comparative analysis of phytochemicals and nutrient availability in two contrasting cultivars of sweet potato (Ipomoea batatas L.). Food Chem. 173, 957–965. 10.1016/j.foodchem.2014.09.172 25466112

[B31] Steed L. Truong V. D. (2008). Anthocyanin content, antioxidant activity, and selected physical properties of flowable purple-fleshed sweetpotato purees. J. Food Sci. 73, S215–S221. 10.1111/j.1750-3841.2008.00774.x 18577013

[B32] Tanaka M. Takahata Y. Kurata R. Nakayama H. Yoshinaga M. (2012). Structural and functional characterization of IbMYB1 genes in recent Japanese purple-fleshed sweetpotato cultivars. Mol. Breed. 29, 565–574. 10.1007/s11032-011-9572-z

[B33] Wang A. Li R. Ren L. Gao X. Zhang Y. Ma Z.. (2018a). A comparative metabolomics study of flavonoids in sweet potato with different flesh colors (*Ipomoea batatas* (L.) Lam). Food Chem. 260, 124–134. 10.1016/j.foodchem.2018.03.125 29699652

[B34] Wang F. Ji G. Xu Z. Feng B. Zhou Q. Fan X.. (2021). Metabolomics and transcriptomics provide insights into anthocyanin biosynthesis in the developing grains of purple wheat (*Triticum aestivum* L.). J. Agric. Food Chem. 69, 11171–11184. 10.1021/acs.jafc.1c01719 34529412

[B35] Wang H. Fan W. Li H. Yang J. Huang J. Zhang P. (2013). Functional characterization of dihydroflavonol-4-reductase in anthocyanin biosynthesis of purple sweet potato underlies the direct evidence of anthocyanins function against abiotic stresses. PLoS ONE. 8, e78484. 10.1371/journal.pone.0078484 24223813 PMC3817210

[B36] Wang H. Wang C. Fan W. Yang J. Appelhagen I. Wu Y.. (2018b). A novel glycosyltransferase catalyses the transfer of glucose to glucosylated anthocyanins in purple sweet potato. J. Exp. Bot. 69, 5444–5459. 10.1093/jxb/ery305 30124996 PMC6255700

[B37] Wang S. Nie S. Zhu F. (2016). Chemical constituents and health effects of sweet potato. Food Res. Int. 89, 90–116. 10.1016/j.foodres.2016.08.032 28460992

[B38] Wei Z. Hu K. Zhao D. Tang J. Huang Z. Jin P.. (2020). MYB44 competitively inhibits the formation of the MYB340-bHLH2-NAC56 complex to regulate anthocyanin biosynthesis in purple-fleshed sweet potato. BMC Plant Biol. 20, 258. 10.1186/s12870-020-02451-y 32503504 PMC7275474

[B39] Xu W. Shioiri H. Kojima M. Nozue M. (2001). Primary structure and expression of a 24-kD vacuolar protein (VP24) precursor in anthocyanin-producing cells of sweet potato in suspension culture. Plant Physiol. 125, 447–455. 10.1104/pp.125.1.447 11154352 PMC61025

[B40] Yamada T. Matsuda F. Kasai K. Fukuoka S. Kitamura K. Tozawa Y.. (2008). Mutation of a rice gene encoding a phenylalanine biosynthetic enzyme results in accumulation of phenylalanine and tryptophan. Plant Cell. 20, 1316–1329. 10.1105/tpc.107.057455 18487352 PMC2438470

[B41] Yi D. Zhang H. Lai B. Liu L. Pan X. Ma Z.. (2021). Integrative analysis of the coloring mechanism of red longan pericarp through metabolome and transcriptome analyses. J. Agric. Food Chem. 69, 1806–1815. 10.1021/acs.jafc.0c05023 33332135

[B42] Yu Y. Wang Y. Yu Y. Ma P. Jia Z. Guo X.. (2021). Overexpression of IbPAL1 promotes chlorogenic acid biosynthesis in sweetpotato. Crop J. 9, 204–215. 10.1016/j.cj.2020.06.003

[B43] Zhang D. Tan Y. Dong F. Zhang Y. Huang Y. Zhou Y.. (2021). The expression of IbMYB1 is essential to maintain the purple color of leaf and storage root in sweet potato [*Ipomoea batatas* (L.) Lam]. Front. Plant Sci. 12, 688707. 10.3389/fpls.2021.688707 34630449 PMC8495246

[B44] Zhang L. Yu Y. Shi T. Kou M. Sun J. Xu T.. (2020). Genome-wide analysis of expression quantitative trait loci (eQTLs) reveals the regulatory architecture of gene expression variation in the storage roots of sweet potato. Hortic. Res. 7, 90. 10.1038/s41438-020-0314-4 32528702 PMC7261777

[B45] Zhang Q. Wang L. Liu Z. Zhao Z. Zhao J. Wang Z.. (2020). Transcriptome and metabolome profiling unveil the mechanisms of Ziziphus jujuba Mill. peel coloration. Food Chem. 312, 125903. 10.1016/j.foodchem.2019.125903 31901700

[B46] Zhang R. Tang C. Jiang B. Mo X. Wang Z. (2021). Optimization of HS-SPME for GC-MS analysis and its application in characterization of volatile compounds in sweet potato. Molecules 26, 5808. 10.3390/molecules26195808 34641353 PMC8510106

[B47] Zhang W. Cao J. Li Z. Li Q. Lai X. Sun L.. (2021). HS-SPME and GC/MS volatile component analysis of Yinghong No. 9 dark tea during the pile fermentation process. Food Chem. 357, 129654. 10.1016/j.foodchem.2021.129654 33866239

[B48] Zhang Y. Fang B. (2006). Dectiptors and Data Standard for Sweetpotato [Ipomoea batatas (L.) Lam.]. Beijing: China Agriculture Press.

[B49] Zhou W. Gong Y. Lu X. Huang C. Gao F. (2012). Molecular cloning and characterization of a flavonoid 3′-hydroxylase gene from purple-fleshed sweet potato (Ipomoea batatas). Mol. Biol. Rep. 39, 295–302. 10.1007/s11033-011-0738-x 21603861

[B50] Zhou W. Huang C. Gong Y. Feng Q. Gao F. (2009). Molecular cloning and expression analysis of an ANS gene encoding anthocyanidin synthase from purple-fleshed sweet potato [*Ipomoea batatas* (L.) Lam]. Plant Mol. Biol. Rep. 28, 112. 10.1007/s11105-009-0133-0

[B51] Zhou W. Zhao S. Xu M. Niu Y. Nasier M. Fan G.. (2021). Identification of key genes controlling carotenoid metabolism during apricot fruit development by integrating metabolic phenotypes and gene expression profiles. J. Agric. Food Chem. 69, 9472–9483. 10.1021/acs.jafc.1c00496 34347458

[B52] Zhu L. Mu T. Ma M. Sun H. Zhao G. (2022). Nutritional composition, antioxidant activity, volatile compounds, and stability properties of sweet potato residues fermented with selected lactic acid bacteria and bifidobacteria. Food Chem. 374, 131500. 10.1016/j.foodchem.2021.131500 34772572

