# Supplementary material for: Combining Metabolomics and Transcriptomics to Reveal the Mechanism of Coloration in Purple and Cream Mutant of Sweet Potato (Ipomoea batatas L.)
Source: Front Plant Sci. 2022 May 4;13:877695. doi: 10.3389/fpls.2022.877695 (PMC9116297; doi:10.3389/fpls.2022.877695)
Supplement: Supplementary file 1 [file Table_1.docx]

Table S1 Primer sequences used for expression analysis of sweet potato genes in this study.

| **Gene name** | **Gene ID** | **Forward** | **Reverse** |
| --- | --- | --- | --- |
| ***IbActin*** | AY905538 | CTGGTGTTATGGTTGGGATGG | GGGGTGCCTCGGTAAGAAG |
| ***IbMYB1-2a/b*** | AB576766.1/ AB576767.1 | GTGGAAAACATGTGCAGTGTCATC | GGACCATGAACCTTTTCTCACTCT |
| ***PAL-1*** | TRINITY_DN71627_c5_g8 | GGGAGCAAGATCAGCCATTG | TTCTCTCCGGTCAGCATCTC |
| ***PAL-2*** | TRINITY_DN62758_c0_g1 | CGAACCTGATCCCGGAGTAG | ACCTCTCATCGACGGACAAA |
| ***PAL-3*** | TRINITY_DN62996_c0_g1 | CAATCATGTCCAGAGTGCCG | AGTGCAGGCCTTCAGATTCT |
| ***C4H*** | TRINITY_DN66972_c5_g18 | TGGGTAATTGGTGGGGTTTT | TGGACATGTACTTTTGCGGC |
| ***4CL-1*** | TRINITY_DN64546_c0_g1 | CGTTGATTCCCATGCTCCAG | TGGGAGGGGAGTTCTACAGA |
| ***4CL-2*** | TRINITY_DN70094_c0_g2 | ACGATTTGAGCTCCATCCGG | CGCCGGACACATTGACAATA |
| ***CHS*** | TRINITY_DN63879_c3_g1 | CCAAAGAAGGCGCCATGTAT | AGCACCTACCCCGACTACTA |
| ***CHI*** | TRINITY_DN64352_c4_g7 | TTTCCGACACCTTCTCCGAA | CGTGTACTTGGAAGCTGACG |
| ***F3‘H*** | TRINITY_DN72050_c5_g4 | AAACCGCACCAGTCTTTAGC | CAATATGTTTGGCGCCGGAG |
| ***F3H-1*** | TRINITY_DN54002_c4_g1 | GTCCGCATTCTTGAACCTCC | CCTGGAACCATCACCCTTCT |
| ***F3H-2*** | TRINITY_DN55749_c0_g2 | CCTGTCAAAGTAGGCGGGT | AGGCTAGGGTTGCATGAATT |
| ***DFR-1*** | TRINITY_DN25583_c0_g1 | ACCTCGTTGTTGTCCTTTGC | AATCCCAACATGTGCCCCTA |
| ***DFR-2*** | TRINITY_DN36881_c0_g2 | CGTCCATGCAACCGTTAGAG | GGCCACATGAAACACACCTT |
| ***ANS*** | TRINITY_DN67033_c16_g25 | CCGTTGTGGTTAATGCCCTC | TGCAGGGCTATGGGAGTAAG |
| ***BZ1-1*** | TRINITY_DN70947_c4_g10 | GAATTCGGGAAGATCGTGCC | TCCAGAGTCGAATTCCACCC |
| ***BZ1-2*** | TRINITY_DN70947_c4_g16 | GGTCATGGCAGAAGGTGAAC | CGAGCGCCTAACCAATTTCT |
| ***3GGT*** | TRINITY_DN71320_c3_g11 | TTCTTCCCCTTTCTCCACCT | CAGCAATTGTTTCTCCAGCA |
| ***IbMYB1*** | TRINITY_DN49283_c0_g1 | AACGTCGGACTTGCTAATGG | TCCCACCACTTCACATTGTC |
| ***R2R3 MYB*** | TRINITY_DN27624_c0_g1 | TGTGGCGTTGTTTCTAGTGC | CGACGACTCTATTGACCGGA |
| ***MYB44*** | TRINITY_DN45230_c0_g1 | TATCAACGCCGGAGAGAGTC | TCATCACATCCCAGAACCCC |
