# Supplementary material for: Combining Metabolomics and Transcriptomics to Reveal the Mechanism of Coloration in Purple and Cream Mutant of Sweet Potato (Ipomoea batatas L.)
Source: Front Plant Sci. 2022 May 4;13:877695. doi: 10.3389/fpls.2022.877695 (PMC9116297; doi:10.3389/fpls.2022.877695)
Supplement: Supplementary file 2 [file Table_2.docx]

Table S2 Plant morphological characters of Xuzi201 and M1001.

|  | Character | Xuzi201 | M1001 |
| --- | --- | --- | --- |
| Vine tip | Vine tip pigmentation | Purple | Purple |
|  | Vine tip pubescence | None | None |
| Immature leaf | Immature leaf color | Purple | Purple |
|  | Immature leaf pubescence | None | None |
| Mature leaf | Mature leaf color | Green | Green |
|  | Abaxial leaf vein pigmentation | All veins partially purple | All veins partially purple |
|  | Petiole pigmentation | Green with purple at both ends | Green with purple at both ends |
|  | Mature leaf length | 10.7±0.67cm | 12.06±0.87cm |
|  | Mature leaf width | 10.2±0.32cm | 11.1±0.51cm |
|  | Petiole length | 14.56±2.24cm | 12.8±1.41cm |
|  | Petiole diameter | 4.72±0.26cm | 3.66±0.44cm |
|  | Mature leaf shape | Cordate | Cordate |
|  | Type of leaf lobes | None lateral lobes | None lateral lobes |
|  | Mature leaf pubescence | None | None |
| Vine | Predominant color of vine | Green with many purple spots | Green with many purple spots |
|  | Secondary color of vine | Purple base and tip | Purple base and tip |
|  | Number of base branches | 4-6 | 3-6 |
|  | Longest vine | 100.66±7.4cm | 94.72±5.3cm |
|  | Vine diameter | 8.43±0.3cm | 7.74±0.16cm |
|  | Vine internode length | 3.4±0.74cm | 2.9±0.5cm |
|  | Twining | Moderately twining | Moderately twining |
|  | Plant type | Spreading | Spreading |
| Root | Skin color | Purple | Cream |
|  | Flesh color | Purple | Cream |
