# Supplementary figures and images for: Combining Metabolomics and Transcriptomics to Reveal the Mechanism of Coloration in Purple and Cream Mutant of Sweet Potato (Ipomoea batatas L.)
Source: Front Plant Sci. 2022 May 4;13:877695. doi: 10.3389/fpls.2022.877695 (PMC9116297; doi:10.3389/fpls.2022.877695)

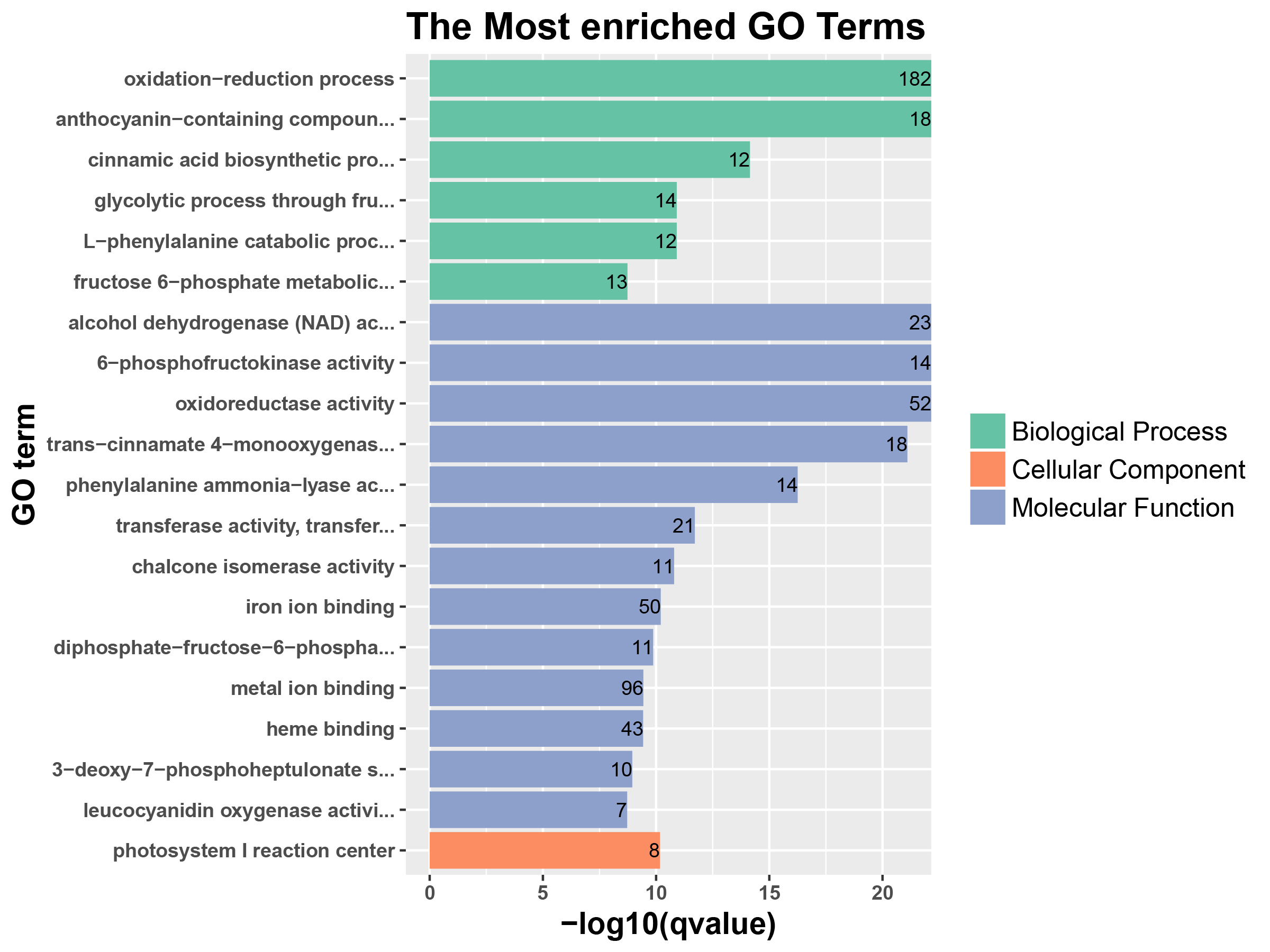

Supplement: Supplementary file 10 [file Image_1.JPEG]

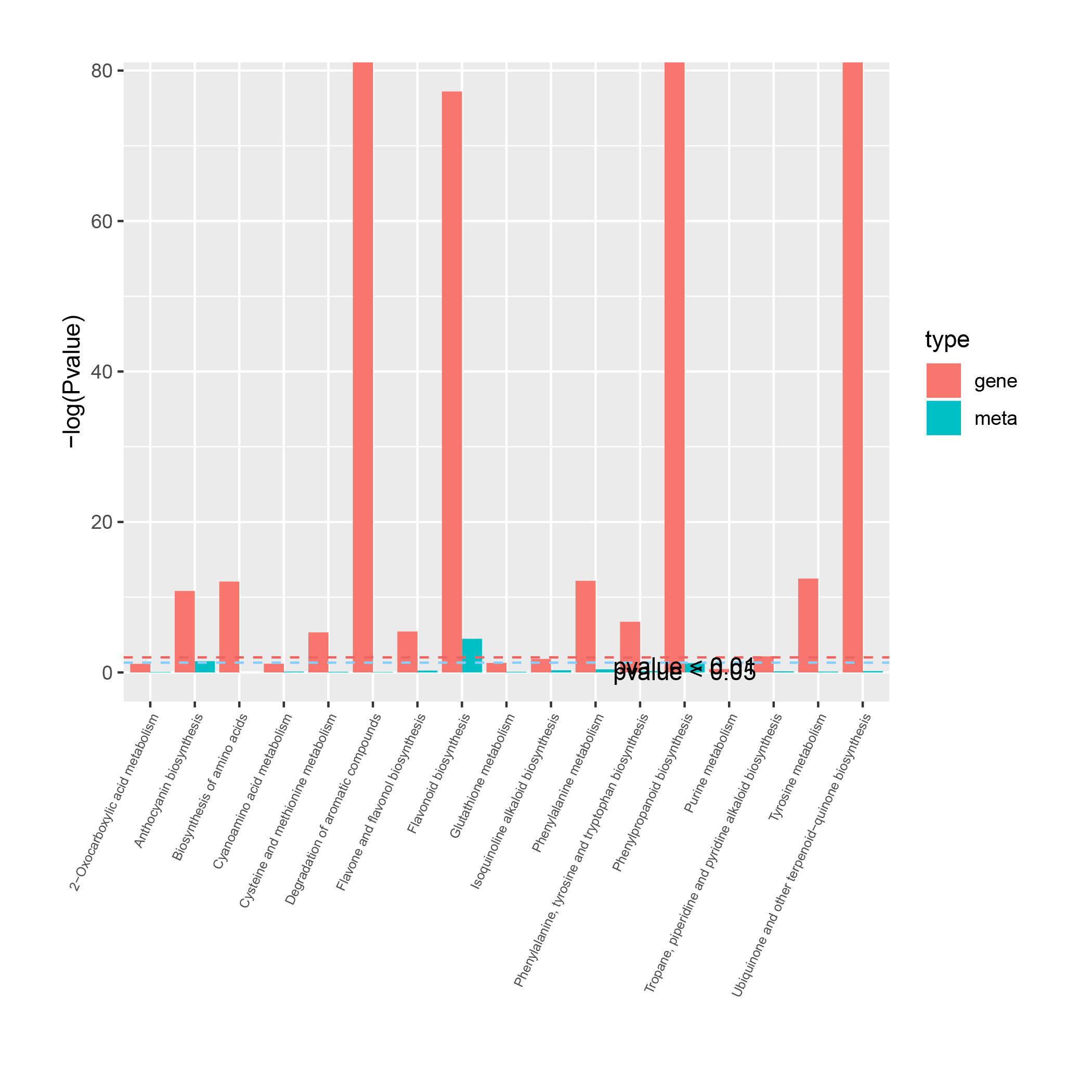

Supplement: Supplementary file 11 [file Image_2.JPEG]

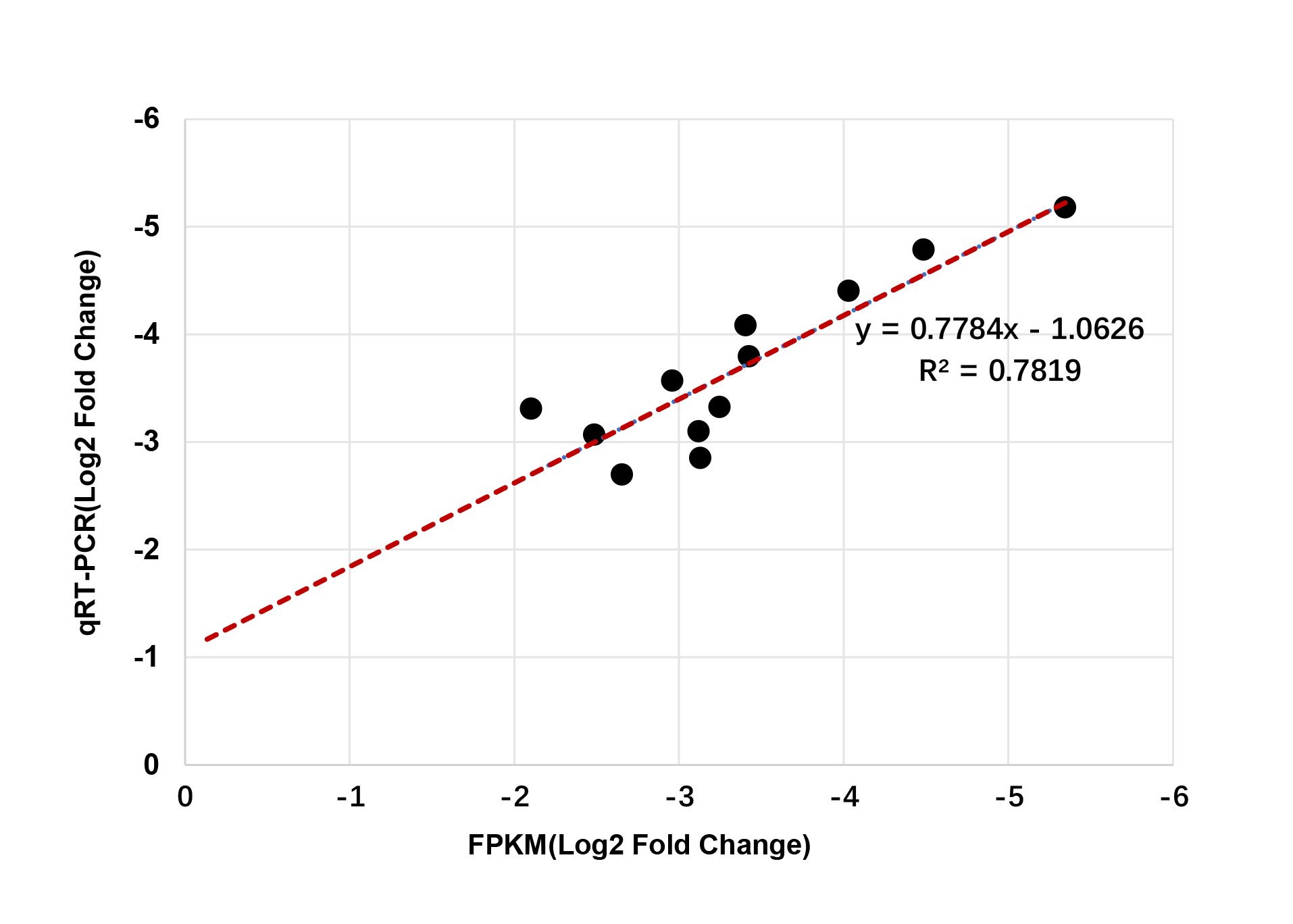

Supplement: Supplementary file 12 [file Image_3.TIF]
